# Super Secondary Structure Consisting of a Polyproline II Helix and a β-Turn in Leucine Rich Repeats in Bacterial Type III Secretion System Effectors

**DOI:** 10.1007/s10930-018-9767-9

**Published:** 2018-04-12

**Authors:** Dashdavaa Batkhishig, Khurelbaatar Bilguun, Purevjav Enkhbayar, Hiroki Miyashita, Robert H. Kretsinger, Norio Matsushima

**Affiliations:** 1 Laboratory of Bioinformatics and Systems Biology, Department of Information and Computer Science, School of Engineering and Applied Sciences, National University of Mongolia, Ulaanbaatar, 14201 Mongolia; 2 Department of Physics, School of Mathematics and Natural Sciences, Mongolian National University of Education, Ulaanbaatar, 210648 Mongolia; 3 Institute of Physics and Technology, Mongolian Academy of Sciences, Enkhtaivan avenue 54B, Ulaanbaatar, 210651 Mongolia; 4 Hokubu Rinsho Co., Ltd, Sapporo, 060-0061 Japan; 5 Institute of Tandem Repeats, Sapporo, 004-0882 Japan; 6 Department of Biology, University of Virginia, Charlottesville, 22904 USA; 7 Sapporo Medical University, Sapporo, 060-8556 Japan

**Keywords:** Bacterial leucine rich repeat family, Polyproline II helix, Type I β-turn, Super secondary structure, Helical parameters, Helix axis, Vector analysis

## Abstract

Leucine rich repeats (LRRs) are present in over 100,000 proteins from viruses to eukaryotes. The LRRs are 20–30 residues long and occur in tandem. LRRs form parallel stacks of short β-strands and then assume a super helical arrangement called a solenoid structure. Individual LRRs are separated into highly conserved segment (HCS) with the consensus of LxxLxLxxNxL and variable segment (VS). Eight classes have been recognized. Bacterial LRRs are short and characterized by two prolines in the VS; the consensus is xxLPxLPxx with Nine residues (N-subtype) and xxLPxxLPxx with Ten residues (T-subtype). Bacterial LRRs are contained in type III secretion system effectors such as YopM, IpaH3/9.8, SspH1/2, and SlrP from bacteria. Some LRRs in decorin, fribromodulin, TLR8/9, and FLRT2/3 from vertebrate also contain the motifs. In order to understand structural features of bacterial LRRs, we performed both secondary structures assignments using four programs—DSSP-PPII, PROSS, SEGNO, and XTLSSTR—and HELFIT analyses (calculating helix axis, pitch, radius, residues per turn, and handedness), based on the atomic coordinates of their crystal structures. The N-subtype VS adopts a left handed polyproline II helix (PPII) with four, five or six residues and a type I β-turn at the *C*-terminal side. Thus, the N-subtype is characterized by a super secondary structure consisting of a PPII and a β-turn. In contrast, the T-subtype VS prefers two separate PPIIs with two or three and two residues. The HELFIT analysis indicates that the type I β-turn is a right handed helix. The HELFIT analysis determines three unit vectors of the helix axes of PPII (**P**), β-turn (**B**), and LRR domain (**A**). Three structural parameters using these three helix axes are suggested to characterize the super secondary structure and the LRR domain.

**Electronic supplementary material:**

The online version of this article (10.1007/s10930-018-9767-9) contains supplementary material, which is available to authorized users.

## Introduction

Leucine rich repeats (LRRs) are unusually rich in leucine [1–6]. The LRRs are composed of 20–30 residues stretches and repeat in tandem. The published repeat numbers range from 2 to 97. LRRs have been reported in over 100,000 proteins from viruses to eukaryotes.

LRR units are divided into a highly conserved segment (HCS) and a variable segment (VS) [1]. Eight classes of LRRs have been recognized [3]. Matsushima and Kretsinger recently proposed twenty-three types of LRRs [1]. Their grouping is based mainly on the difference of the VS parts. The eight classes are RI-like, cysteine containing (CC), SDS22-like, IRREKO, bacterial, plant specific, typical, and TpLRR.

The HCS part consists of an 11 or 12 residue stretch, LxxLxLxx(N/C)(x/-)L, in which “L” is Leu, Ile, Val, or Phe, “N” is Asn, Thr, Ser, or Cys, “C” is Cys, Ser or Asn, “x” is any amino acid, and “−” is a deletion. Three residues at positions 3–5 in the underlined residues form a short β-strand [4, 6]. These β-strands stack parallel; they have the pattern of H-bonding (N–H → O=C), and then these tandem repeats of LRRs assume their super helical arrangements. The LRRs fold into a horse shoe, a right handed or left handed helix, or a prism shape [7]. Conserved hydrophobic residues such as leucine, valine, isoleucine, or phenylalanine in the consensus sequences of LRRs contribute to the hydrophobic cores. Capping structures that shield the hydrophobic core of the first LRR unit at the *N*-terminus and/or the last unit at the *C*-terminus are observed in most of the known LRR structures [1, 5].

Characteristic of each LRR class, the VS parts adopt a variety of secondary structures including the α-helix, 3_10_-helix, and an extended conformation or a tandem arrangement of β-turns [1, 6]. Their secondary structures on the convex side are connected to the strands forming the β-sheet on its concave side by two loops [5]. One of the loops is an “ascending loop” which links the *C*-terminus of the HCS to the *N*-terminus of the VS. The other is a “descending loop” which links the *C*-terminus of the VS to the *N*-terminus of the HCS of the following unit. Each LRR domain contains a concave surface, a convex surface, an ascending surface, and a descending surface on the opposite side. LRR domains are involved in direct interaction with proteins (including hormones) or ligands (including nucleic acid, lipid, lipo-polysaccharide, and plant steroid hormones) [1]. LRR domains can engage structurally various proteins or ligands using different surfaces of the LRR domains [1, 5].

LRR proteins participate in the plant immune response and in the mammalian innate immune response [1–6]. They are also involved in a broad range of functions including apoptosis, autophagy, ubiquitin related processes, nuclear mRNA transport, and neuronal development [1, 8]. Plant LRR proteins, many of which involve kinases and other receptor like proteins, act as signal amplifiers in tissue damage, in symbiotic relationships, and in developmental processes [1, 9].

Furthermore, LRR proteins are contained in the type III secretion system of many gram-negative bacterial pathogens. The LRR proteins called effectors are delivered into the cytosol of animal or plant cells [10]. Consequently, these effectors enable the bacteria to avoid the immune response of the infected organism by modulating cell functions of the host. The effector proteins include YopM from the bubonic plague bacterium, *Yersinia pestis*, and SspH1, SspH2, and SlrP from *Salmonella enterica*, and IpaH3 and IpaH9.8 from *Shigella flexneri*. These effectors are bacterial LRR proteins [1]. The LRR domain of SspH1 directly interacts with PKN1 [11].

Bacterial LRRs are characterized by two Leu Pro sequences in the VS; the consensus is xxLPxLPxx with Nine residues (N-subtype) and xxLPxxLPxx with Ten residues (T-subtype) where “L” is Leu, Val, or Ile and “x” is predominantly occupied by small residues such as Thr, Ser, or Gly. T-subtype is seen in *Salmonella* SlrP. Moreover, LRRs in the subfamily of toll-like receptors (TLR7, TLR8, and TLR9), the small leucine rich repeat proteoglycan (SLRP) family including fibromodulin, decorin and biglycan, and the fibronectin leucine rich repeat transmembrane family (FLRT) contains N- or T-subtype [1]. The LRRs consist of tandem repeats of a super motif of *STT* or *ST* in which “*S*” is Bacterial and “*T*” is Typical [12–14]. We called this the *STT* class [1].

PPIIs are known to be observed frequently in proline rich regions [15–19]. The PPIIs are characterized by the backbone dihedral angles (Φ, Ψ) of (− 75°, 145°) [20–29]. The PPIIs have helical parameters: 2.9 residues per turn, a pitch value of 8.7 Å per turn, and a helix radius of 1.33 Å. The assignment of PPII is not done in the widely used programs such as DSSP [28] and STRIDE [29]. Consequently, PPIIs in newly solved protein structures are not registered in protein data bank (PDB) [30]. Now there are some tools for assigning PPII number—DSSP-PPII [31], PROSS [32], SEGNO [33], XTLSSTR [34], and ASSP [35].

Super secondary structures with several adjacent elements of a secondary structure are also observed in protein structures [36, 37]. Examples include β-hairpins, α-helix hairpins, and β–α–β motifs. Adzhubei and Sternburg [38] identified super secondary structures consisting of PPII and α-helix and of 3_10_-helix and PPII. Kumar and Bansal [27] also identified those consisting of β-strand and PPII, of β-strand, PPII, and α-helix, and of β-strand, PPII, and β-strand.

Evdokimov et al. [39] noted that the VS parts in the YopM LRRs adopt 3_10_-helices. Matsushima et al. [6, 7], Bella et al. [5], and Park et al. [40] proposed that the VS parts in the bacterial LRR adopt left handed polyproline II helices (PPII). A review article by Adzhubei et al. [41] noted PPIIs in LRRs. However, it appears that PPII in LRR structures has not yet been well characterized based on the consensus sequence. Structural data of proteins containing bacterial LRRs have increased. The crystal structures of YopM, SspH1, SspH2, SlrP, IpaH3, and IpaH9.8 have been determined [39, 42–46]. The structures of TLR8, TLR9, fibromodulin, decorin, biglycan, FLRT2, and FLRT3 are also available [47–56].

The purpose of this study is to understand structural features of bacterial LRRs. We performed both the secondary structures analyses using secondary structures prediction programs of DSSP-PPII, PROSS, SEGNO, and XTLSSTR and the HELFIT analyses that calculate helix axis, helix pitch, helix radius, repeat/residue number per turn, based on the atomic coordinates of the crystal structures [57].

This present analysis demonstrates that the N-subtype VS adopts PPII consisting of 4–6 residues and type I β-turn at the *C*-terminal side. Thus, the VS part is characterized by super secondary structure consisting of PPII and a β-turn. In contrast, the T-subtype VS frequently prefers two separate PPIIs consisting of two or three and of two residues. The HELFIT analysis indicates that the type I β-turn is a right handed helix and consequently determines three unit vectors of the helix axes of PPII (**P**), β-turn (**B**), and LRR domain (**A**). We propose three structural parameters which are two angles between the two helix axes of PPII and β-turn, between the two helix axes of PPII and LRR domain, and between the helix axis of LRR domain and the vector product of **P** × **B**. These three angles are suggested to characterize the super secondary structure and the LRR domain.

## Methods

### Structure Data

We collected the structure data of proteins containing bacterial LRRs from the PDB. We performed sequence alignments in LRR proteins from the PDB by LRRpred that recognizes and aligns LRR motifs that predict the repeat number and “phasing” of LRRs with greater reliability [58] and identified bacterial LRR based on the consensus sequence. Bacteria LRR proteins are YopM, SspH1, SspH2, SlrP, IpaH3, IpaH9.8, TLR8, TLR9, fibromodulin, decorin, biglycan, FLRT2, and FLRT3 (Table 1) [39, 42–56]. LRRs in YopM, SspH1, SspH2, SlrP, IpaH3, and IpaH9.8 belong to bacterial LRR class, while TLR8, TLR9, fibromodulin, decorin, biglycan, FLRT2, and FLRT3 belong to the *STT* class (Table 1). Eighteen PDB files solved at resolution ≤ 3.4 Å were used; the sequence identity of the 18 different chains shows that the maximum is 48% and the average is 7% (Supplementary Table S1). The structure data of mouse FLRT2 at resolution 4.0 and 6.0 Å were not used for analyses.

**Table 1:** Known structures of proteins containing bacterial LRRs

Number of protein	LRR class	Protein name	Repeat number of LRR^a^	Number of N-subtype	Number of T-subtype	PDBID	Chains	Resolution (Å)
1	Bacterial	*Y. pestis* yopM	16 (16)	13	0	1JL5	A	2.10
	Bacterial	*Y. pestis* yopM	16 (16)	13	0	1G9U	A	2.35
2	Bacterial	*Y. entercocolitica* yopM	21 (20)	19	0	4OW2	A,B,C,D	3.20
3	Bacterial	*S. enterica* SspH1	10 (9)	6	1	4NKH	A,B,C,D,E,F	2.75
	Bacterial	*S. enterica* SspH1	10 (9)	6	1	4NKG	A,C	2.90
4	Bacterial	*S. enterica* SspH2	13 (13)	10	1	3G06	A	1.90
5	Bacterial	*S. flexneri* ipaH3	9 (9)	5	1	3CVR	A	2.80
6	Bacterial	*S. flexneri* ipa9.8	8 (8)	5	1	5B0N	A,B	1.80
	Bacterial	*S. flexneri* ipa9.8	8 (8)	5	1	5B0T	A	2.00
7	Bacterial	*S. enterica* SlrP	12 (12)	0	10	4PUF	A,B	3.30
8	STT	Human fibromodulin	13 (12)	2	2	5MX0	A,B	2.21
9	STT	Horse TLR9	27 (27)	1	1	3WPC	A,B	1.60
	STT	Horse TLR9	27 (27)	1	1	3WPB	A	2.40
	STT	Horse TLR9	27 (27)	1	1	3WPD	A	2.75
10	STT	Bovine TLR9	27 (27)	1	1	3WPE	A	2.38
11	STT	Mouse TLR9	27 (27)	1	1	3WPF	A	1.96
	STT	Mouse TLR9	27 (27)	1	1	3WPG	A	2.25
	STT	Mouse TLR9	27 (27)	1	1	3WPI	A	2.25
	STT	Mouse TLR9	27 (27)	1	1	3WPH	A	2.35
12	STT	Human TLR8	27 (27)	0	3	3WN4	A	1.81
	STT	Human TLR8	27 (27)	0	3	3W3J	A,B	2.00
	STT	Human TLR8	27 (27)	0	3	3W3N	A,B	2.10
	STT	Human TLR8	27 (27)	0	3	3W3G	A,B	2.30
	STT	Human TLR8	27 (27)	0	3	3W3K	A,B	2.30
	STT	Human TLR8	27 (27)	0	3	3W3L	A,B,C,D	2.33
	STT	Human TLR8	27 (27)	0	3	3W3M	A	2.70
13	STT	Bovine decorin	12 (12)	0	3	1XKU	A	2.15
	STT	Bovine decorin	12 (12)	0	3	1XEC	A,B	2.30
	STT	Bovine decorin	12 (12)	0	3	1XCD	A	2.31
14	STT	Bovine biglycan	12 (12)	0	3	2FT3	A,B,C,D,E,F	3.40
15	STT	Human TLRT2	13 (13)	0	3	4V2D	A	2.50
16	STT	Mouse TLRT2	13 (13)	0	3	5FTT	B,F	3.40
17	STT	Human TLRT3	13 (13)	0	3	5CMP	A,B,C,D	2.60
18	STT	Mouse FLRT3	13 (13)	0	3	4V2E	A,B	2.50
	STT	Mouse FLRT3	12 (12)	0	3	2YEB	B,F	3.19

^a^The number in the parentheses indicates the number of variable segment of LRRs

### Secondary Structures Analysis

Secondary structures assignments were made from the atomic coordinates of the LRR structures using four programs—DSSP-PPII [31], PROSS [32], SEGNO [33], and XTLSSTR [34]. The assignment of the DSSP program is based on the identification of precise hydrogen bond patterns corresponding to regular secondary structures [28]. In DSSP-PPII based on DSSP, PPII are assigned solely in the coil region for at least two consecutive residues in coil with Φ = − 75° ± 29° and Ψ = +145° ± 29°. The PROSS program assigns secondary structures, based mainly on Φ and Ψ dihedral angles. SEGNO utilizes the Φ and Ψ dihedral angles coupled with other angles. XTLSSTR uses two angles and three distances. DSSP-PPII, PROSS, and XTLSSTR assign β-turns; while SEGNO does not. The secondary assignments were performed using the PolyprOnline web interface [59]. Types of β-turn were also identified by the programs of PROMOTIF [60] and STRIDE [61]. Furthermore, the root-mean-square deviation (RMSD) of the VS part between within the N-subtype and within the T-subtype and between these two subtypes using the coordinates of the backbone atoms of each residue were evaluated by the CHIMERA program [62].

### HELFIT Analysis

We have developed a total least squares program for fitting a helix to data points—HELFIT [57]. A helix consisting of *n* repeat units may be characterized by helix axis, helix pitch (*P*), helix radius (*R*), and number of repeat units/residue per turn (*N*). HELFIT computes these parameters in which the helix axis is represented by the unit vector. These parameters also yield the rise per repeat unit/residue (Δ*z* = *P*/*N*) and the rotation per repeat unit/residue in the helix (ΔΦ = 360°/*N*). Moreover, HELFIT gives *rmsd*: where *d*_i_ is the closest distance from the data point to the trace of the helix.

1 $$rmsd={\left[ {{{\left( {{\text{the}}\,{\text{minimum of }}\sum {{d_{\text{i}}}} } \right)} \mathord{\left/ {\vphantom {{\left( {{\text{the}}\,{\text{minimum of }}\sum {{d_{\text{i}}}} } \right)} N}} \right. \kern-0pt} N}} \right]^{{1 \mathord{\left/ {\vphantom {1 2}} \right. \kern-0pt} 2}}}$$

Here *p* = *rmsd* / (*n* − 1)^1/2^ gives the regularity of helical structures independent of the number of data points or helix length. The criterion for regular PPII helices is *p* ≤ 0.10 Å. This same test is used for α-helices, ω-helices, and 3_10_-helices in proteins [63, 64]. The HELFIT analysis requires only four data points: the coordinates of α-carbon (Cα) of each residue. LRRs form a β-strand of three residues at positions 3–5 in the HCS part. Thus, in LRRs, the C_α_ coordinates of the consensus leucine residue at position four in individual LRR repeat units are used. The repeat number of individual LRR domains was defined as the number that participates in the parallel β-sheet. This definition means that the first LRR is sometimes contained in the capping structures. β-Turns consist of four amino acid residues (labelled *i, i* + 1, *i* + 2, and *i* + 3). We also estimate the helical parameters of β-turns using the C_α_ coordinates of each residue.

The HELFIT analysis indicates that the β-turn is regarded as a right handed helix, as noted later. Consequently, HELFIT determines three unit vectors of the helix axes of LRR domain (**A**), PPII (**P**), and type I β-turn (**B**). We estimate three structural parameters. One is the angle between the two helix axes of PPII and β-turn (Ω_1_). The second is the angle between the two helix axes of PPII and LRR domain (Ω_2_). The third is the angle between the helix axis of LRR domain and the vector product of **P** × **B**. (Ω_3_). The three angles of Ω_1_, Ω_2_, and Ω_3_ are represented by the following equations.

2 $${\mathbf{P}} \cdot {\mathbf{B}}=\left| {\mathbf{P}} \right|\left| {\mathbf{B}} \right|\cos {\Omega _1}$$

3 $${\mathbf{P}} \cdot {\mathbf{A}}=\left| {\mathbf{P}} \right|\left| {\mathbf{A}} \right|\cos {\Omega _2}$$

4 $$({\mathbf{P}} \times {\mathbf{B}}) \cdot {\mathbf{A}}=\left| {{\mathbf{P}} \times {\mathbf{B}}} \right|\left| {\mathbf{A}} \right|\cos {\Omega _3}$$

## Results

### Two Subtypes of Consensus Sequences

**Fig. 1:**
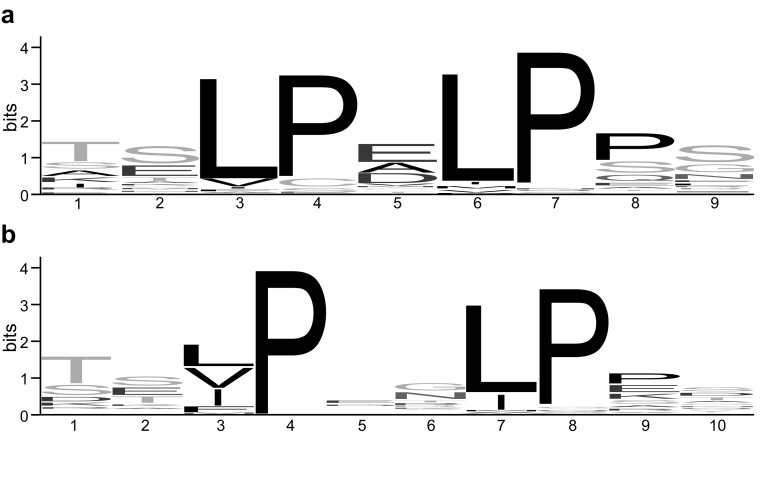
Consensus sequence of variable segment (VS) of bacterial LRRs in the known structures. **a** N-subtype; **b** T-subtype. The graphical sequence diagrams were generated with WebLogo [78], representing sixty-three LRR units for the N-subtype and forty-three for the T-subtype

Bacterial LRR is 20 or 21 residues long and is classified into two subtypes. The N-subtype has the VS consensus of xxLPxLPxx with nine residues and the T-subtype has xxLPxxLPxx with ten residues where “L” is Leu, Val, or Ile and “x” is predominantly occupied by small residues such as Thr, Ser, or Gly (Fig. 1). Thus bacterial LRR is characterized by two Leu Pro sequences in the VS parts; although variable VS that lack one of the two conserved prolines is observed. The characteristics are not seen in other LRR classes. For examples, ribonuclease inhibitors and Nod-like receptors contain RI-like LRRs which of the consensus is LxxLxLxx(N/C)xLxxxgoxxLxxoLxxzxxx with typically 28 or 29 residues [65].

The N-subtype appears sixty-three times in the known structures (Table 1). The VS consensus is xxLPxLPxx, as expected, in which “x” positions at the *N*- and *C*-terminal sides are frequently occupied by relatively small residues such as Thr or Ser; while the central “x” position is rich in Glu. Fifty-two of the sixty-three VS are completely consistent with the consensus (Fig. 1a). The remaining VSs are xxL**x**xLPxx in YopM; the conserved Pro at position four is replaced by Cys or Ser. The T-subtype appears forty-three times (Table 1). The conserved Leu at position three in the VS consensus is frequently occupied by other hydrophobic residues such as Val or Ile (Fig. 1b).

### Secondary Structures

The assignment of PPII patterns differs among the four programs. Bacterial LRR proteins form not only monomers but also homo-dimers, -tetramers, and -hexamers in crystals (Table 1). The PPII patterns assigned also differ among their individual molecules. We therefore analyzed all chains of the known structures.

The four programs for secondary structures assignment indicate that the HCS parts adopt short β-strands in three underlined residues of Lx**xLx**LxxNxL. In addition, the assignment indicates that the VS parts are rich in PPII conformations. However, the secondary structures show a difference between the two subtypes.

**Fig. 2:**
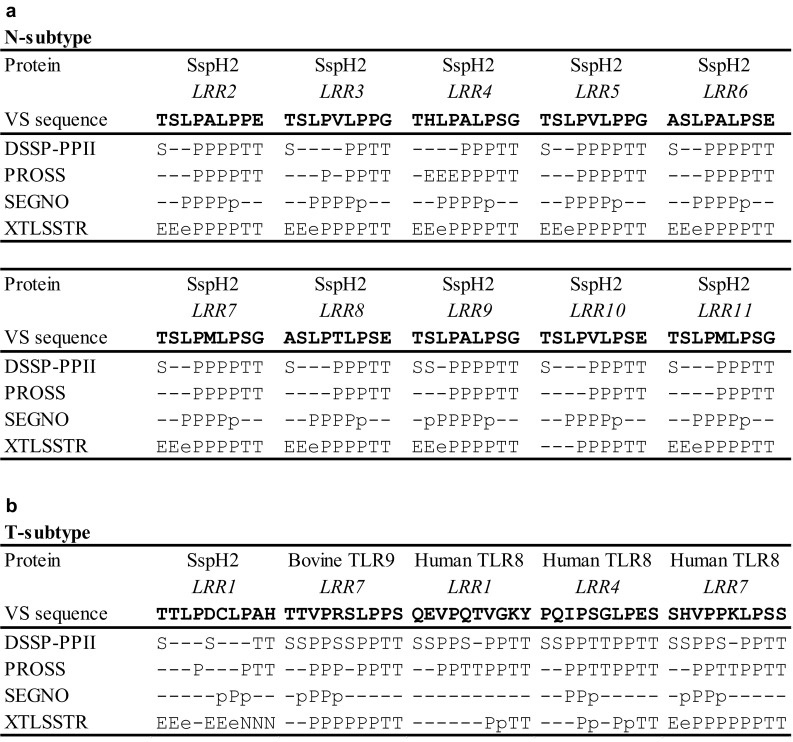
Secondary structure assignment of the variable segment (VS) of representative bacterial LRRs by the four programs (DSSP-PPII, PROSS, SEGNO, and XLTSSTR). **a** N-subtype, SspH2 (PDB ID: 3G06_A). **b** T-subtype, SspH2 (*LRR1*) (PDB ID: 3G06_A), Bovine TLR9 (*LRR7*) (PDB ID:3WPC_A) and Human TLR8 (*LRR1, LRR4*, and *LRR7*) (PDB ID: 3WN4_A). A one letter code is used to represent a specific conformation; P and p, PPII; E and e, β-strand; T, β-turn; N, non-hydrogen-bonded β-turn; H, α-helix; and S, bend

At least one of the four programs of secondary structures assignment indicates that the N-subtype VS adopts PPIIs consisting of four, five or six residues (Fig. 2a; Supplementary Table S2). Four, five or six residue PPIIs are observed in the underlined residues of xxL**PxLP**xx, xx**LPxLP**xx, and x**xLPxLP**xx, respectively. For example, all four program assign PPII in underlined residues of KK**LPDLP**LS (*LRR7*) in *Y. pestis* YopM (*n* = 12) [3G06_A] (Fig. 2a). The variable VS of xxL**xxxLP**xx frequently adopt four or five PPIIs. The HELFIT analysis demonstrated that all of the PPIIs assigned are definitely left handed polyproline helices, as noted later.

In addition, the programs identified β-turns at the *C*-terminal side in the VS parts. The sequences in the underlined residues of xxLPxLP**xx**LxxLxLxxNxL adopt β-turns; the second conserved Pro corresponds to the residue, *i*, of β-turns. The types are distinguished by the Φ, Ψ, angles of residues *i* + 1 and *i* + 2. The average Cα (*i*)−Cα (*i* + 3) is 5.48 (0.22 Å); the numbers in parenthesis are standard deviations; they are reasonable [66]. The Φ, Ψ angles of residues *i* + 1 and *i* + 2 of the β-turns have the average angles of Φ_*i*+1_ = − 60.7 (8.8°), Ψ_*i*+1_ = − 22.6 (8.4°), Φ_*i*+2_ = − 94.0 (13.8°), Ψ_*i*+2_ = 3.4 (14.0°); the numbers in parentheses are standard deviations. These values are close to − 60°, − 30° and − 90°, 0° which define the type I β-turns. The β-turns assignments by PROMOTIF [60] and STRIDE [61] give the same results and indicate that most of the β-turns are type I (Table 2). Types IV and VIII rarely appear. Also very rarely β-turns are not assigned.

**Table 2:** Types of β-turns at the *C*-terminal side in the bacterial LRRs

	DSSP-II	PROSS	XLTSSTR
Type I	66	87	137
Type IV	5	5	5
Type VIII	1	0	1
Total	72	92	143

In the T-subtype VS PPIIs assigned may be divided into three patterns. Many VSs adopt two separate PPIIs with two or three and two residues, which are observed in the underlined residues of xx**LP**xx**LP**xx and x**xLP**xx**LP**xx (Fig. 2b; Supplementary Table S2). The second is one PPIIs with three or four residues in the underlined residues of xx**LPx**xLPxx, xxLPx**xLP**xx or x**xLPx**xLPxx. The third pattern is six residue PPII in the underlined residues of xx**LPxxLP**xx, which are seen in decorin, TLR9, and TLR8 by the XLTSSTR program. Moreover, the *C*-terminal two residues of the T-subtype VS are assigned to adopt mostly type I β-turn as does that of the N-subtype VS.

The average RMSD of the VS parts of the N-subtypes and of the T-subtypes is 0.589 (0.315 Å) and 0.993 (0.337 Å), respectively; all Bacterial VSs show the RMSD of 1.087 (0.549 Å).

**Fig. 3:**
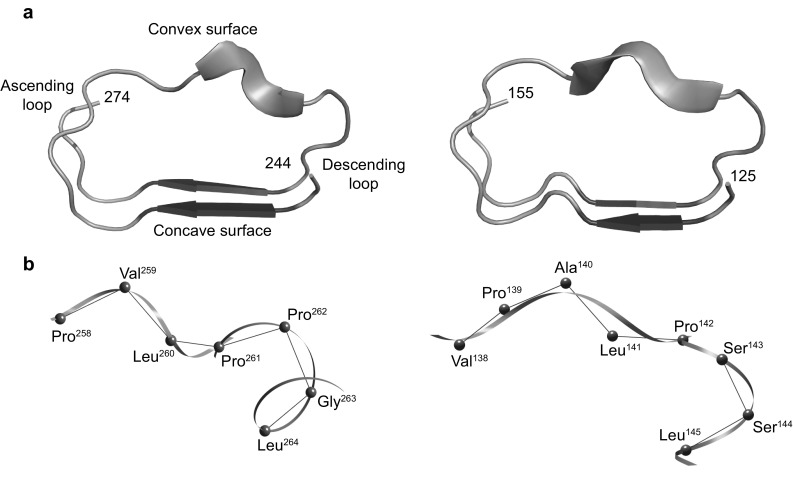
Super secondary structure consisting of a PPII and a β-turn in bacterial LRRs. **a** Secondary structures. Left panel. Sequence 244-LRTLEVSGNQLTSLPVLPPGLLELSIFSNPL-274 in SspH2 (*LRR3* and HCV of *LRR4*) (PDB ID: 3G06_A). Right panel. 125-LEELNLSYNGITTVPALPSSLVSLILSRTNI-155 in bovine TLR9 (*LRR4* and HCS of *LRR5*) (PDB ID:3WPC_A). Blue arrows represent β-strands, green ribbons PPIIs, and red tubes β-turns. **b** Super secondary structure (HELFIT). Left panel. Sequence 258-PVLPPGL-264 in SspH2 (PDB ID: 3G06_A). The sequence PVLP is a part of the *LRR3* VS which correspond to the underlined residues of xxLPxLPxx (the consensus of the N-type VS. The sequence PVLP adopt PPII and the PPGL adopt type I β-turn. The sequence PVLP is a part of the LRR3 VS which correspond to the underlined residues of xxLPxLPxx (the consensus of the N-type VS). The sequence PVLP adopts four residue PPII and the PPGL adopts type I β-turn. Right panel. Sequence 138-VPALPSSL-145 in bovine TLR9 (PDB ID: 3WPC_A). The sequence VPALP is a part of the *LRR4* VS which correspond to the underlined residues of xxLPxLPxx. The sequence VPALP forms five residue PPII and the sequence PPSL forms one type I *β*-turn. Best fitted lines by HELFIT are colored green for PPIIs and red for β-turns, and α-carbons grey. (Color figure online)

In conclusion the N-subtype VS is characterized by a super secondary structure consisting of PPII with four, five, or six residues and a type I β-turn (Fig. 3), while the T-subtype VS strongly prefers one or two separate PPIIs and adopts a type I β-turn at the C-terminal side as does the N-subtype VS. It appears that the structure of the T-subtype is more variable.

### Helical Parameters of PPIIs, Type I β-turns, and LRR Domains

The number of assigned PPII of which the helix length is longer than three residues increase in order of DSSP-PPII < PROSS < XLTSSTR < SEGNO (Table 3). SEGNO assigns longer PPIIs. Four residue PPIIs are regular and thus are a near ideal form (Table 2). All of five and six residue PPIIs are irregular. The helix regularity decreases with increasing helix length. The deviation of helix parameters from ideal values increases with increasing helix length. The helix irregularities in the five and six residue PPIIs are mostly due to larger deviations of the Φ, Ψ angles from ideal: − 75°, 145° in the first and/or second residues of N-terminal side in the sequence of **xL**PxLP. The sequence of VP(A/R)LP in TLR9 (LRR4), which corresponds to the underlined residues of xx**LPxLP**xx, adopt five residue PPIIs with large helix irregularity; *p* = 0.41–0.47 Å. In this case, the irregularity comes from large deviation of the Φ, Ψ angles of the conserved Leu at the *C*-terminal side. Taking account of the helical parameters, the five residue PPIIs are regarded as a highly deformed form. Consequently, the HELFIT analysis demonstrated that all four, five, and six residue PPIIs assigned by the secondary structures analyses are regular or irregular left handed polyproline helices.

**Table 3:** Helix parameters of PPIIs in bacterial LRRs

	Programs	Number of PPII	*P* (Å)^a^	*N* ^a^	*R* (Å)^a^	Δ*z* (Å)^a^	*p* (Å)	*V*_c_ (Å^3^)^a^
PPII^b^	‒	‒	8.96	2.99	1.36	3.00	‒	17.41
PPII^c^	‒	‒	8.69	2.90	1.33	3.00	‒	16.70
PPII^d^	‒	‒	8.58	2.99	1.45	2.87	‒	18.95
PPII^e^	‒	‒	8.62	3.08	1.52	2.80	‒	20.30
Four residue PPII	DSSP-PPII	50	8.61 (0.40)	2.82 (0.13)	1.25 (0.05)	3.06 (0.05)	0.10 (0.03)	15.07 (1.07)
	PROSS	67	8.52 (0.51)	2.78 (0.21)	1.23 (0.09)	3.07 (0.08)	0.10 (0.04)	14.70 (1.74)
	SEGNO	21	8.78 (0.31)	3.05 (0.10)	1.47 (0.05)	2.87 (0.05)	0.05 (0.04)	19.49 (0.05)
	XLTSSTR	130	8.45 (0.38)	2.75 (0.15)	1.23 (0.07)	3.07 (0.07)	0.10 (0.04)	14.49 (1.49)
Five residue PPII	DSSP-PPII	49	9.83 (0.50)	3.54 (0.25)	1.53 (0.11)	2.78 (0.10)	0.26 (0.04)	20.50 (2.39)
	PROSS	32	9.94 (0.43)	3.64 (0.16)	1.58 (0.06)	2.73 (0.05)	0.27 (0.03)	21.46 (1.39)
	SEGNO	132	10.05 (0.37)	3.65 (0.15)	1.57 (0.07)	2.75 (0.07)	0.27 (0.04)	21.38 (1.62)
	XLTSSTR	43	10.17 (1.22)	3.54 (0.39)	1.47 (0.16)	2.88 (0.18)	0.29 (0.09)	19.39 (3.10)
Six residue PPII	SEGNO	21	10.45 (1.21)	3.82 (0.50)	1.50 (0.09)	2.74 (0.07)	0.30 (0.04)	19.56 (2.18)
	XLTSSTR	14	13.91 (1.64)	6.10 (0.71)	2.40 (0.09)	2.28 (0.05)	0.45 (0.04)	41.36 (2.92)

The number in the parentheses indicates standard deviations

^a^*P* helix pitch, *N* residue number per turn, *R* helix radius, Δ*z* helix rise per turn; Vc = πR^2^(Δ*z*)

^b^(Φ, Ψ) = (− 75, 150) by Jha [67]

^c^(Φ, Ψ) = (− 75, 145) by Hopfinger [25]

^d^(Φ, Ψ) = (− 65, 140) by Adzhubei [68]

^e^(Φ, Ψ) = (− 60, 140) by Schulz and Schirmer [26]

In the T-subtype only the XLTSSTR program identifies long PPIIs with six residues in *LRR7* of horse/bovine TLR9, *LRR4* of human TLR8, and *LRR4* of decorin, as noted. The *p* values are very large; *p* = 0.38–0.50 Å (Table 3). The high irregularity comes from the Φ, Ψ angle of any residue at position six in the T-subtype VS consensus of xxLPx**x**LPxx; the Φ, Ψ angles are in regions of a left handed α-helix. In addition, the helical parameters deviate highly from those of ideal form (Table 3). The six residue PPIIs assigned are not recognized as a PPII. Alternatively, the SEGNO program identifies regular, four residue PPIIs in the underlined residues of x**xLPx**xLPxx (Supplementary Table S2). Thus, the six residue PPIIs may be divided into two separate PPIIs with three and two residues. This supports the conclusion by the secondary structure assignment that the T-subtype VS contains two separate PPIIs.

The average helix parameters of the type I β-turns are; *P* = 6.11→ 6.14 Å, *N* = 3.65 →3.68 residues/turn, *R* = 2.26 →2.28 Å, and Δ*z* = 1.68 → 1.69 Å (Table 4). It appears that the helical parameters are close to those of α-helix as it has *P* = 5.4 Å, *N* = 3.6 residues/turn, *R* = 2.4 Å, and Δ*z* = 1.5 Å. The average *p* value is 0.02→ 0.03 Å. The HELFIT analysis indicates that these type I β-turns form a regular, right handed helix.

**Table 4:** Helix parameters of β-turn and three structural parameters in bacterial LRRs

Programs	Number of secondary structure	Helical parameters of the β-turns					Structural parameters			
		*P* (Å)^a^	*N* ^a^	*R* (Å)^a^	Δ*z* (Å)^a^	*p* (Å)	*V*_c_ (Å^3^)^a^	Ω_1_ (°)^b^	Ω_2_^b^ (°)^b^	Ω_3_ (°)^b^
DSSP-PPII	72	6.14 (0.32)	3.68 (0.23)	2.28 (0.15)	1.68 (0.16)	0.03 (0.02)	16.36 (2.16)	99.8 (9.0)	30.6 (6.9)	100.6 (8.2)
PROSS	92	6.11 (0.34)	3.66 (0.22)	2.26 (0.14)	1.68 (0.15)	0.03 (0.02)	26.90 (1.65)	101.3 (10.2)	30.8 (7.1)	99.9 (7.6)
XLTSSTR	143	6.14 (0.31)	3.65 (0.22)	2.26 (0.14)	1.69 (0.14)	0.03 (0.02)	26.93 (1.58)	103.7 (8.1)	33.4 (6.5)	99.3 (8.9)

The number in the parentheses indicates standard deviations

^a^*P* helix pitch, *N* residue number per turn, *R* helix radius; Δ*z* helix rise per turn; *V*_c_ = π*R*^2^(Δ*z*)

^b^Ω_1_ is the angle between the two helixes of the PPII and the β-turn; Ω_2_ is the angle between the two helixes of the PPII and the LRR domain; Ω_3_ is the angle between the helix of the LRR domain and the vector product of **P**×**B** in which **P** and **B** is the unit vectors of the helices of the PPII and the β-turn, respectively

The helix parameters of LRR domains were determined for IpaH9.8 (*n* = 8), SspH1, (*n* = 10), SspH2 (*n* = 13), *Y. pestis* YopM (*n* = 16), *Y. enterocolitica* YopM (*n* = 21), and SlrP (*n* = 12). The bacterial LRR domains are represented by a right handed helix (Table 5). The helix parameters range over: *P* = 47.3 → 115 Å, *N* = 28.4 → 41.3 units/turn, *R* = 18.9 → 24.6 Å, Δ*z* = 1.67 → 3.46 Å, and ΔΦ = 8.7° → 12.7°; *p* = 0.03 → 0.19 Å.

**Table 5:** Helix parameters of LRR domains in bacteria LRR proteins

	Protein	*n* ^a^	*P*(Å)^a^	*N* ^a^	*R*(Å)^a^	Δ*z* (Å)^a^	ΔΦ (°)^a^	*p*(Å)^a^	PDB_chanis
1	IpaH9.8	8	114.94	33.19	18.87	3.46	10.85	0.14	5B0N_A
	IpaH9.8	8	111.48	34.21	20.48	3.26	10.52	0.13	5B0N_B
2	SspH2	13	76.72	30.57	21.57	2.51	11.78	0.03	3G06
3	YopM	16	47.31	28.37	20.58	1.67	12.69	0.08	1JL5A
4	YopM	21	71.27	30.35	21.70	2.35	11.86	0.12	4OW2_A
	YopM	21	67.16	30.46	22.10	2.20	11.82	0.14	4OW2_B
	YopM	21	71.42	29.75	21.09	2.40	12.10	0.08	4OW2_C
	YopM	21	70.50	29.58	20.99	2.38	12.17	0.09	4OW2_D
5	SspH1	10	84.44	34.74	23.69	2.43	10.36	0.14	4NKH_A
	SspH1	10	88.78	35.95	24.31	2.47	10.01	0.13	4NKH_B
	SspH1	10	83.55	35.39	24.63	2.36	10.17	0.13	4NKH_C
	SspH1	10	89.67	34.39	23.14	2.61	10.47	0.16	4NKH_D
	SspH1	10	103.20	32.90	20.47	2.50	10.94	0.16	4NKH_E
	SspH1	10	83.66	41.32	29.48	2.02	8.71	0.19	4NKH_F
	SspH1	10	89.67	33.55	22.10	2.67	10.73	0.16	4NKG_A
	SspH1	10	87.89	30.75	19.83	2.86	11.71	0.15	4NKG_C
6	IpaH3	9	89.22	35.25	24.13	2.53	10.21	0.07	3CVR
7	slrP	12	70.03	34.93	25.34	2.00	10.31	0.11	4PUF_A
	slrP	12	54.07	35.31	26.44	1.53	10.20	0.15	4PUF_B

^a^*n* repeat number of LRRs, *P* helix pitch, *N* residue number per turn, *R* helix radius, Δ*z* helix rise per turn, ΔΦ rotation per repeat unit, *p* helix regularity

**Fig. 4:**
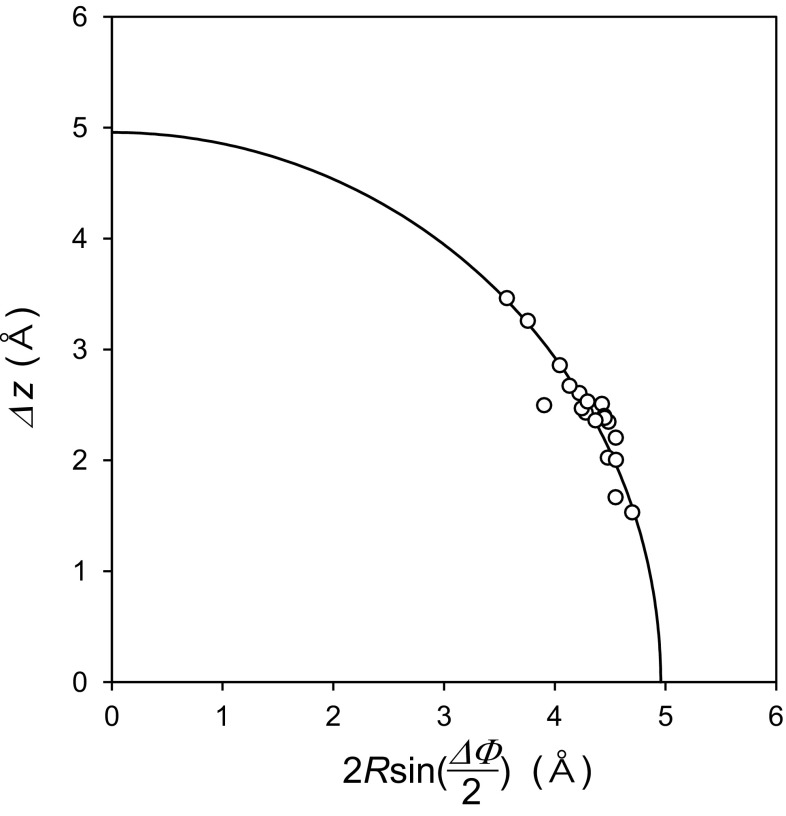
The correlation of Δz and 2·*R*·sin(ΔΦ/2) in the helix parameters of LRR domains in the seven bacterial LRR proteins

Figure 4 shows a plot of 2·*R*·sin (ΔΦ/2) versus Δ*z*. The values fall on a circle with radius *D* (circle plot) [7]. *D* is a function of Δ*z*, ΔΦ, and *R* [7].

5 $$D = \left[ {\left\{ {2R\sin \left( {\frac{{\Delta \Phi }}{2}} \right)} \right\}^{2} + (\Delta z)^{2} } \right]^{{1/2}}$$

*D* is the average C_α_(*i*) – C_α_(*i* + 1) distance between adjacent repeats—*i* and *i* + 1; *D* corresponds to the inter-strand distance. Equation 5 gives *D* = 4.97 ± 0.10 Å; this allows the formation of hydrogen bonds between parallel strands. This circle plot shows that the helix pitch, *P*, and rise per turn, Δ*z*, of bacterial LRR is comparable to those of SDS22-like and Plant specific LRRs; while it is larger than those of RI-like and CC LRRs [7].

### Geometrical Analysis

**Fig. 5:**
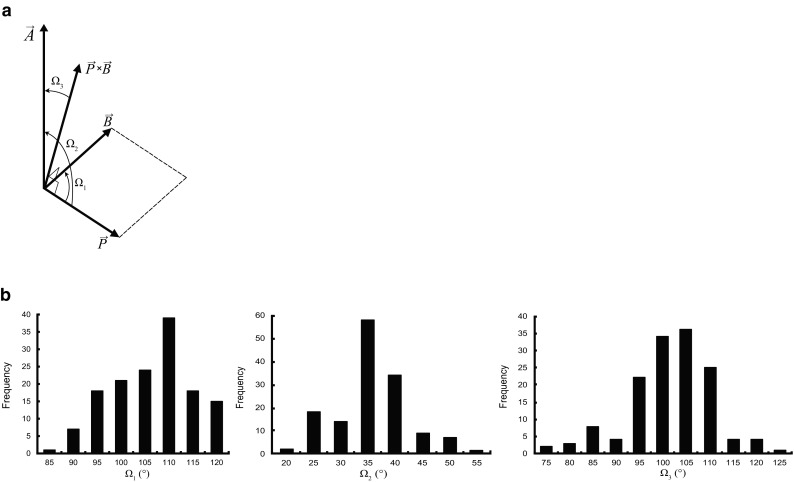
Geometrical analysis of super secondary structure. **a** The definition of the three angles of Ω_1,_ Ω_2_, and Ω_3_. **b** Frequency distributions of the Ω_1,_ Ω_2_, and Ω_3_ angles (by the XLTSSTR program)

Figure 5 shows the frequency distributions of three angles of Ω_1_, Ω_2_, and Ω_3_. The Ω_1_ angle shows an asymmetrical distribution (Fig. 5b). The Ω_1_ angle ranges from 70° to 120°; the average value is ∼ 103° (Table 4). The Ω_2_ and Ω_3_ have the average values of ∼ 33° and ∼ 99°, respectively.

## Discussion

### Structural Role of PPIIs in Bacterial LRRs

The backbone dihedral angles (Φ, Ψ) of ideal PPII is (− 75°, 145°) [23]. Other dihedral angles has been also proposed; (Φ, Ψ) = (− 75°, 140°), (− 65°, 145°), and (− 60°, 140°) (Table 3) [26, 67, 68]. It appears that four residue PPIIs in proteins are a near ideal form with (Φ, Ψ) = (− 75°, 145°) or (− 75°, 140°).

**Fig. 6:**
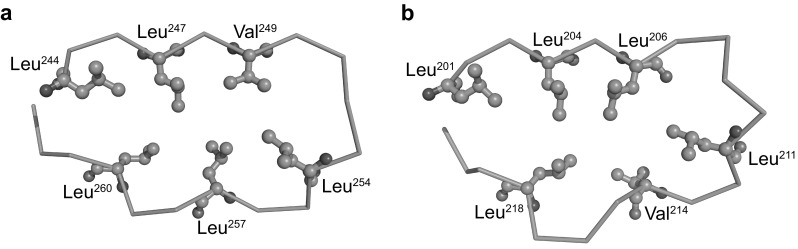
Hydrophobic cores in the N-subtype (**a**) and T-subtype (**b**) of bacterial LRR. **a** Sequence 244-LRTLEVSGNQLTSLPVLPPG-263 (*LRR3*) in SspH2 (PDB ID: 3G06_A). **b** Sequence 211-LTHLSLKYNNLTTVPRSLPPS-221 (*LRR7*) in bovine TLR9 (PDB ID:3WPC_A). Green is α-carbon, red oxygen, and blue nitrogen. (Color figure online)

The secondary structure assignment and the HELFIT analysis indicate that the N-subtype VS adopts one stretch of PPII of four, five, or six residues. In contrast, the T-subtype prefers two separate PPIIs consisting two or three and of two residues. Consequently, there is a clear difference in the PPII patterns between the two subtypes. The two hydrophobic residues in the VS part of the two subtypes are concentrated on the side that is oriented toward the hydrophobic core as well as other conserved hydrophobic residues in the HCS part (Fig. 6). This structural restriction makes the difference. The assignments by the four programs show different PPII patterns in most cases. This observation mainly comes from high flexibility of PPII due to no intrachain hydrogen bond.

The variable N-type VSs are seen in YopM—RSLCDLPPS and SGLSELPPN (Supplementary Table S2). The first repeat of twelve LRRs in chondroadherin is also a variable N-subtype VS of QKIPKVSEK; the structure of human chondroadherin which forms tetramers in crystal has been determined at 2.1 Å resolution [69]. Their secondary structure assignment sometimes shows no PPII or only short PPII of two or three residues. These observations indicate that two conserved prolines in the N-subtype VS parts are strongly required for the super secondary structure consisting of PPII and β-turn.

It may be significant that Src tyrosine kinases SH3 domain binds to short proline rich sequence of xPxxP that forms PPII [70]. This sequence is very similar to LPxLP in the N-subtype.

### Super Secondary Structure

The present analyses demonstrate that the N-subtype adopts a super secondary structure consisting of a PPII and a β-turn. Ananthanarayanan et al. [71] first described this super secondary structure. Tandem repeats of the super secondary structure form a novel helical structure called the polyproline, β-turn helix [72]. This structure is observed in tandem repeats of the hepta-peptide, YSPSPSPS, in the *C*-terminal repeat domain (CTD) of the large subunit of RNA polymerase II (POL II) [73–75]. Many factors involved in RNA processing bind the CTD [76].

The VSs of RI-like and CC LRRs adopt an α-helical conformation (β–α structural units). Typical LRR VSs prefer tandem β-turns. The SDS22-like LRR VSs strongly prefer 3_10_-helix (β-3_10_). The Plant specific LRR structural unit is β-β-3_10_. The occurrence of β-turns at the *C*-terminal sides of the VS parts are also observed in Plant specific, SDS22-like, and Typical LRRs as well as bacterial LRR [7]. Consequently, a super secondary structure consisting of 3_10_-helix and β-turns is present in Plant specific and SDS22-like LRRs. The unique super secondary structures consisting of β-turns and PPII, and of β-turns and 3_10_-helix should be recognized as structural elements in proteins.

Here we propose a structural parameter that characterizes the super secondary structure; the parameter is the angle between the two helix axes of PPII and type I β-turn (Ω_1_). Its average value is ∼ 103°. The Ω_1_ angle also helps to characterize other super secondary structures including those consisting of PPII and an α-helix, and of PPII and a 3_10_-helix.

### Solenoid Structure of Bacterial LRR Domains

The circle plot of bacterial LRR is comparable to those of SDS22-like and Plant specific LRR; while it differs from those of RI-like and CC LRRs (Fig. 4). We recently calculated helical parameters of 642 LRRs of known structures of 114 proteins by the HELFIT program [7]. The results indicate that the helical parameters are influenced by the structures of the ascending loops rather than of the descending loops, helical elements on the convex face, and the uniformity of parallel strand stacking on the concave face [1, 7]. The helix radius of PPII is the smallest between α-helix, 3_10_-helix, π-helix, ω-helix, and PPII. This partly contributes to a relatively large helix pitch for the bacterial LRR domains.

We determined the Ω_2_ angle between the helix axes of PPII and of the bacterial LRR domain. The VSs of SDS22-like, Plant-specific, CC, and RI-like adopt 3_10_-helix or α-helix instead of PPII. The comparison of the Ω_2_ angle with the angles between their helices and LRR domains may identify fundamental features of individual LRR classes.

### The PPII Assignment by the Four Programs

In many methods for assignments of secondary structures from atomic coordinates, the termini of the segments are frequently ill-defined and it is difficult to decide unambiguously which residues at the edge of the segments have to be included [77]. In this study the PPII pattern assigned also differs between the four programs in most cases. The number of four, five, or six residue PPIIs assigned is larger in SEGNO and XLTSSTR than in DSSP-PPII and PROSS. However, the HELFIT analyses indicate that all PPIIs assigned are unambiguously regular or irregular left handed polyproline helices with only a few exceptions. The combination of the secondary structure assignment programs (SEGNO and XLTSSTR) and the HELFIT analysis is useful for PPII assignment in proteins.

## Conclusions

The present study shows that the N-subtype bacterial LRRs are characterized by a unique super secondary structures consisting of PPII helices and a β-turn. In contrast, the T-subtype VS prefers two separate PPIIs with two or three or with only two residues. The type I β-turns can be regarded as regular, right handed helices. We propose three important structural parameters: the three angles between the two helix axes of PPII and β-turn, between two helix axes of PPII and LRR domain, and between the helix axis of LRR domain and the vector product of **P** × **B**. These three angles characterize the super secondary structure and the LRR domain.

### **Funding**

This study was funded by National University of Mongolia (FELLOWSHIP GRANT-P2016-1173) (to P. E.)

## Electronic supplementary material

Below is the link to the electronic supplementary material.

Supplementary material 1. Table S1 Percentage identity of 18 different protein chains containing bacterial LRRs. (PDF 116 KB)

Supplementary material 2. Table S2 Secondary structure assignments of the N-subtype VS and the T-subtype VS of bacterial LRR (PDF 313 KB)

## Abbreviations

LRR: Leucine rich repeat
PPII: Polyproline II helix
HCS: Highly conserved segment
VS: Variable segment
C_α_: α-Carbon
CC: Cysteine containing
*P*: Helix pitch
*R*: Helix radius
*N*: Number of repeat unit/residue per turn in helix
δ*z*: Rise per repeat unit/residue in helix
ΔΦ: Rotation angle per repeat unit/residue in helix
*n*: Repeat number of leucine rich repeat
PDB: Protein data bank
RMSD: Root mean square deviation
TLR: Toll like receptor
SLRP: Small leucine rich repeat proteoglycan protein
FLRT: Fibronectin leucine rich repeat transmembrane protein
CTD: *C*-terminal repeat domain of the large subunit of RNA polymerase II
POL II: RNA polymerase II
