# Supplementary material for: Super Secondary Structure Consisting of a Polyproline II Helix and a β-Turn in Leucine Rich Repeats in Bacterial Type III Secretion System Effectors
Source: Protein J. 2018 Apr 12;37(3):223–36. doi: 10.1007/s10930-018-9767-9 (PMC5976695; doi:10.1007/s10930-018-9767-9)
Supplement: Supplementary file 1 — Supplementary material 1. Table S1 Percentage identity of 18 different protein chains containing bacterial LRRs. (PDF 116 KB) [file 10930_2018_9767_MOESM1_ESM.pdf]

	PDB:Chain	1	2	3	4	5	6	7	8	9	10	11	12	13	14	15	16	17	18
1	1JL5:A	100																	
2	4OW2:A	10.04	100																
3	4NKH:A	9.20	5.80	100															
4	3G06:A	7.07	9.82	12.13	100														
5	3CVR:A	5.42	14.28	2.92	5.78	100													
6	5B0N:A	8.48	12.94	15.62	8.03	8.92	100												
7	5MX0:A	10.49	5.80	5.52	8.83	5.80	4.41	100											
8	3WPC:A	3.74	10.49	7.94	3.74	4.36	5.35	6.62	100										
9	3WPE:A	3.62	10.26	7.94	4.61	3.87	4.91	6.35	48.18	100									
10	3WPF:A	3.98	8.70	7.94	4.73	5.72	5.80	4.69	39.85	42.46	100								
11	4PUF:A	6.27	5.80	2.98	9.57	6.59	9.37	6.62	6.12	4.08	5.33	100							
12	3WN4:A	3.82	7.58	7.94	4.68	6.04	9.82	7.18	8.22	8.61	7.97	5.96	100						
13	1XKU:A	7.27	5.80	7.11	3.85	4.72	8.03	4.69	1.87	1.99	2.86	3.29	2.95	100					
14	2FT3:A	5.42	4.46	5.43	4.18	4.20	5.35	6.35	3.24	3.24	3.61	2.51	2.21	6.02	100				
15	4V2D:A	8.28	6.44	6.74	8.58	4.60	6.25	4.97	7.97	8.28	6.44	4.60	9.81	6.13	10.73	100			
16	5FTT:B	10.32	6.78	7.37	9.73	4.42	5.30	4.69	5.89	5.60	7.07	7.37	5.89	7.66	7.66	8.25	100		
17	5CMP:A	8.40	6.60	5.40	7.80	8.10	4.80	5.24	9.00	9.00	8.70	7.20	6.90	6.30	6.00	3.60	8.55	100	
18	4V2E:A	5.13	8.15	5.13	6.34	6.94	8.03	7.18	5.13	4.53	5.13	8.76	8.45	8.15	6.34	5.43	5.30	5.70	100

Minimum: 2%

Maxxmum: 48%

Average: 7%
