# Supplementary material for: Super Secondary Structure Consisting of a Polyproline II Helix and a β-Turn in Leucine Rich Repeats in Bacterial Type III Secretion System Effectors
Source: Protein J. 2018 Apr 12;37(3):223–36. doi: 10.1007/s10930-018-9767-9 (PMC5976695; doi:10.1007/s10930-018-9767-9)
Supplement: Supplementary file 2 — Supplementary material 2. Table S2 Secondary structure assignments of the N-subtype VS and the T-subtype VS of bacterial LRR (PDF 313 KB) [file 10930_2018_9767_MOESM2_ESM.pdf]

N-subtype

**YopM**	**1G9U_A**	LRR1	LRR2	LRR3	LRR5	LRR7	LRR9				
	**VS sequence**	**SSLPELPPH**	**TELPELPQS**	**KALSDLPPL**	**KKLPDLPPS**	**KKLPDLPLS**	**KTLPDLPPS**				
	DSSP-PPII	S---PPPTT	SS-PP--TT	SB-----TT	S-PPPPPTT	S-PPPPPTT	SS-PPPPTT				
	PROSS	----PPPTT	-EEEP-PTT	-EEEP-PTT	-EEEPPPTT	--PPPPPTT	---PPPPTT				
	SENGO	----PPPp-	-pPPPPp--	--PPPPp--	--PPPPp--	--PPPPp--	--PPPPp--				
	XTLSSTR	----PPPP-	EEePPPPTT	---PPPPNN	EEePPPPTT	--PPPPPTT	EEePPPPTT				
	**1G9U_A**	LRR10	LRR11	LRR12	LRR13	LRR14	LRR15				
	**VS sequence**	**TDLPELPQS**	**SGLSELPPN**	**RSLCDLPPS**	**IELPALPPR**	**AEVPELPQN**	**REFPDIPES**				
	DSSP-PPII	S---PPPTT	S----PPTT	----PPPTT	SSPPPPPTT	S---PPPTT	SSPP-PPTT				
	PROSS	---PPPPTT	-EEE-PPTT	----PPPTT	-EEEPPPTT	--PPPPPTT	SSPP-PPTT				
	SENGO	-pPPPPp--	---pPPp--	----PPp--	-pPPPPp--	--PPPPp--	-pPPpPp--				
	XTLSSTR	EEePPPPTT	---PPPPTT	EEePPPPTT	EEePPPPTT	--PPPPPTT	--PPPPPTT				
**YopM**	**1JL5_A**	LRR1	LRR2	LRR3	LRR5	LRR7	LRR9				
	**VS sequence**	**SSLPELPPH**	**TELPELPQS**	**KALSDLPPL**	**KKLPDLPPS**	**KKLPDLPLS**	**KTLPDLPPS**				
	DSSP-PPII	S---SPPTT	SS-PPPPTT	S---SPPTT	S--PPPPTT	SS-PPPPTT	SS--SPPTT				
	PROSS	----PPPTT	---PPPPTT	---PPPPTT	---PPPPTT	---PPPPTT	---PPPPTT				
	SENGO	--PPPPp--	--PPPPp--	--PPPPp--	--PPPPp--	--PPPPp--	--PPPPp--				
	XTLSSTR	EEePPPPTT	EEePPPPTT	EEePPPPTT	EEePPPPTT	EEePPPPTT	EEe--PpTT				
	**1JL5_A**	LRR10	LRR11	LRR12	LRR13	LRR14	LRR15				
	**VS sequence**	**TDLPELPQS**	**SGLSELPPN**	**RSLCDLPPS**	**IELPALPPR**	**AEVPELPQN**	**REFPDIPES**				
	DSSP-PPII	S---PPPTT	SEES---TT	SEE-PPPTT	S---PPPTT	S-PPPPPTT	SSPPP--TT				
	PROSS	---PPPPTT	------PTT	---PPPPTT	--PPPPPTT	--PPPPPTT	--PPP-PTT				
	SENGO	--PPPPp--	---------	--PPPPp--	--PPPPp--	--PPPPp--	--PPP-PTT				
	XTLSSTR	EEePPPPTT	---EEeTTT	EEePPPPTT	---PPPPTT	EEePPPPTT	--PPPPPTT				
**YopM**	**4OW2_A**	LRR1	LRR2	LRR3	LRR5	LRR6	LRR7	LRR8	LRR9	LRR10	LRR11
	**VS sequence**	**SSLPELPPH**	**TELPELPQS**	**KALPDLPPL**	**KTLPDLPPS**	**TALPELPQS**	**SGLSELPPN**	**RSLCDLPPS**	**IELPALPSG**	**AELPELPPN**	**SSLCDLPPS**
	DSSP-PPII	S---PPPSS	SS-PPPPTT	S---PPPTT	S-PPPPPTT	----PPPTT	-------TT	----PPPTT	S---PPPTT	S---PPPTT	S------TT
	PROSS	----PPPTT	-EEEPPPTT	---PPPPTT	--PPPPPTT	---PPPPTT	------PTT	--P-PPPTT	---PPPPTT	--PPPPPTT	--P-PPPTT
	SENGO	--PPPPPp-	--PPPPp--	--PPPPp--	--PPPPp--	--PPPPp--	---------	--PPPPp--	--PPPPp--	--PPPPp--	--PPPPp--
	XTLSSTR	EEePPPPP-	EEePPPPTT	--PPPPPTT	EEePPPPNN	EEePPPPTT	------NNN	EEePPPPTT	EEePPPPTT	EEePPPPTT	S-PPPPPTT
	**4OW2_A**	LRR12	LRR13	LRR14	LRR15	LRR16	LRR17	LRR18	LRR19	LRR20	
	**VS sequence**	**IELPALPPD**	**AELPELPPN**	**SSLCDLPPS**	**IELPALPPD**	**AELPELPPN**	**SSLCDLPPS**	**IELPALPPH**	**AEVPELPQN**	**REFPDIPES**	
	DSSP-PPII	S---PPPTT	S-PPPPPTT	----PPPTT	S---PPPTT	S-PPPPPTT	--PPPPPTT	SB-PPPPTT	SBPPPPPTT	SSPPPPPTT	
	PROSS	-EEEPPPTT	--PPPPPTT	---PPPPTT	--P-PPPTT	--PPPPPTT	--PPPPPTT	---PPPPTT	--PPPPPTT	--PPP-PTT	
	SENGO	--PPPPp--	--PPPPp--	---pPPp--	--PPPPp--	--PPPPp--	--PPPPp--	--PPPPp--	--PPPPp--	-pPbBb---	

	XTLSSTR	EEePPPPTT	--PPPPPTT	Eee-PPpTT	EEePPPPTT	EEePPPPTT	EEePPPPTT	EEePPPPTT	---PPPPTT	--PPPPPTT	
**YopM**	**4OW2_B**	LRR1	LRR2	LRR3	LRR5	LRR6	LRR7	LRR8	LRR9	LRR10	LRR11
	**VS sequence**	**SSLPELPPH**	**TELPELPQS**	**KALPDLPPL**	**KTLPDLPPS**	**TALPELPQS**	**SGLSELPPN**	**RSLCDLPPS**	**IELPALPSG**	**AELPELPPN**	**SSLCDLPPS**
	DSSP-PPII	S---PPPSS	S--PPPPTT	S---PPPTT	S-PPPPPTT	----PPPTT	-S---PPTT	----PPPTT	S---PPPTT	S---PPPTT	S---PPPTT
	PROSS	----PPPTT	-EEEPPPTT	---PPPPTT	--PPPPPTT	---PPPPTT	---P-PPTT	--P-PPPTT	---PPPPTT	--PPPPPTT	--P-PPPTT
	SENGO	--PPPPPp-	--PPPPp--	--PPPPp--	--PPPPp--	--PPPPp--	-bBbPPp--	--PpPPp--	--PPPPp--	--PPPPp--	--PPPPp--
	XTLSSTR	EEePPPPP-	EEePPPPTT	--PPPPPTT	EEePPPPNN	EEePPPPTT	EEePPPPNN	--PPPPPTT	EEePPPPTT	--PPPPPTT	--PPPPPTT
	**4OW2_B**	LRR12	LRR13	LRR14	LRR15	LRR16	LRR17	LRR18	LRR19	LRR20	
	**VS sequence**	**IELPALPPD**	**AELPELPPN**	**SSLCDLPPS**	**IELPALPPD**	**AELPELPPN**	**SSLCDLPPS**	**IELPALPPH**	**AEVPELPQN**	**REFPDIPES**	
	DSSP-PPII	S---PPPTT	S-PPPPPTT	----PPPTT	S---PPPTT	S-PPPPPTT	--PPPPPTT	SB-PPPPTT	SB-PPPPTT	SSPPPPPTT	
	PROSS	-EEEPPPTT	--PPPPPTT	---PPPPTT	--P-PPPTT	--PPPPPTT	--PPPPPTT	---PPPPTT	---PPPPTT	--PPP-PTT	
	SENGO	--PPPPp--	--PPPPp--	---pPPp--	--PPPPp--	--PPPPp--	--PPPPp--	--PPPPp--	--PPPPp--	-pPbBb---	
	XTLSSTR	--PPPPPTT	--PPPPPTT	EEe-PPpTT	EEePPPPTT	EEePPPPTT	EEePPPPTT	EEePPPPTT	---PPPPTT	--PPPPPTT	
**YopM**	**4OW2_C**	LRR1	LRR2	LRR3	LRR5	LRR6	LRR7	LRR8	LRR9	LRR10	LRR11
	**VS sequence**	**SSLPELPPH**	**TELPELPQS**	**KALPDLPPL**	**KTLPDLPPS**	**TALPELPQS**	**SGLSELPPN**	**RSLCDLPPS**	**IELPALPSG**	**AELPELPPN**	**SSLCDLPPS**
	DSSP-PPII	S---PPPSS	S--PPPPTT	S---PPPTT	S-PPPPPTT	----PPPTT	-S---PPTT	-----PPTT	S---PPPTT	S---PPPTT	S---PPPTT
	PROSS	----PPPTT	-EEEPPPTT	---PPPPTT	--PPPPPTT	---PPPPTT	---P-PPTT	-----PPTT	---PPPPTT	--PPPPPTT	--P-PPPTT
	SENGO	--PPPPPp-	--PPPPp--	--PPPPp--	--PPPPp--	--PPPPp--	-bBbPPp--	---------	--PPPPp--	--PPPPp--	--PPPPp--
	XTLSSTR	EEePPPPP-	EEePPPPTT	--PPPPPTT	EEePPPPNN	EEePPPPTT	EEePPPPNN	---EEeTTT	EEePPPPTT	EEePPPPTT	--PPPPPTT
	**4OW2_C**	LRR12	LRR13	LRR14	LRR15	LRR16	LRR17	LRR18	LRR19	LRR20	
	**VS sequence**	**IELPALPPD**	**AELPELPPN**	**SSLCDLPPS**	**IELPALPPD**	**AELPELPPN**	**SSLCDLPPS**	**IELPALPPH**	**AEVPELPQN**	**REFPDIPES**	
	DSSP-PPII	S---PPPTT	S-PPPPPTT	----PPPTT	S---PPPTT	S-PPPPPTT	--PPPPPTT	SB-PPPPTT	SBPPPPPTT	SSPPPPPTT	
	PROSS	-EEEPPPTT	--PPPPPTT	---PPPPTT	--P-PPPTT	--PPPPPTT	--PPPPPTT	---PPPPTT	--PPPPPTT	--PPP-PTT	
	SENGO	--PPPPp--	--PPPPp--	---pPPp--	--PPPPp--	--PPPPp--	--PPPPp--	--PPPPp--	--PPPPp--	-pPbBb---	
	XTLSSTR	EEePPPPTT	--PPPPPTT	EEe-PPpTT	EEePPPPTT	EEePPPPTT	EEePPPPTT	EEePPPPTT	---PPPPTT	--PPPPPTT	
**YopM**	**4OW2_D**	LRR1	LRR2	LRR3	LRR5	LRR6	LRR7	LRR8	LRR9	LRR10	LRR11
	**VS sequence**	**SSLPELPPH**	**TELPELPQS**	**KALPDLPPL**	**KTLPDLPPS**	**TALPELPQS**	**SGLSELPPN**	**RSLCDLPPS**	**IELPALPSG**	**AELPELPPN**	**SSLCDLPPS**
	DSSP-PPII	S---PPPSS	SS-PPPPTT	S---PPPTT	S-PPPPPTT	----PPPTT	-S---PPTT	-----PPTT	S---PPPTT	S---PPPTT	S---PPPTT
	PROSS	----PPPTT	-EEEPPPTT	---PPPPTT	--PPPPPTT	---PPPPTT	---P-PPTT	-----PPTT	---PPPPTT	--PPPPPTT	--P-PPPTT
	SENGO	--PPPPPp-	--PPPPp--	--PPPPp--	--PPPPp--	--PPPPp--	-bBbPPp--	---------	--PPPPp--	--PPPPp--	--PPPPp--
	XTLSSTR	EEePPPPP-	EEePPPPTT	--PPPPPTT	EEePPPPNN	EEePPPPTT	EEePPPPNN	---EEeTTT	EEePPPPTT	EEePPPPTT	--PPPPPTT
	**4OW2_D**	LRR12	LRR13	LRR14	LRR15	LRR16	LRR17	LRR18	LRR19	LRR20	
	**VS sequence**	**IELPALPPD**	**AELPELPPN**	**SSLCDLPPS**	**IELPALPPD**	**AELPELPPN**	**SSLCDLPPS**	**IELPALPPH**	**AEVPELPQN**	**REFPDIPES**	
	DSSP-PPII	S---PPPTT	S-PPPPPTT	----PPPTT	S---PPPTT	S-PPPPPTT	--PPPPPTT	SB-PPPPTT	SBPPPPPTT	SSPPPPPTT	
	PROSS	-EEEPPPTT	--PPPPPTT	---PPPPTT	--P-PPPTT	--PPPPPTT	--PPPPPTT	---PPPPTT	--PPPPPTT	--PPP-PTT	
	SENGO	--PPPPp--	--PPPPp--	---pPPp--	--PPPPp--	--PPPPp--	--PPPPp--	--PPPPp--	--PPPPp--	-pPbBb---	

	XTLSSTR	--PPPPPTT	--PPPPPTT	EEe-PPpTT	EEePPPPTT	EEePPPPTT	EEePPPPTT	EEePPPPTT	---PPPPTT	--PPPPPTT
**SspH1**	**4NKH_A**	LRR2	LRR3	LRR4	LRR5	LRR6	LRR7			
	**VS sequence**	**TSLPELPEG**	**TSLPSLPQG**	**ASLPTLPPG**	**TSLPEMPPA**	**TSLPALPSG**	**TSLPEMSPG**			
	DSSP-PPII	S---PP-TT	---PPPPTT	S--PPPPTT	S--PPPPTT	---PPPPTT	S---PPPTT			
	PROSS	---PPP-TT	---PPPPTT	---PPPPTT	---PPPPTT	---PPPPTT	---PPPPTT			
	SENGO	--PPPPp--	--PPPPp--	--PPPPp--	--PPPP---	--PPPPp--	--PPPPp--			
	XTLSSTR	---PPPPTT	EEePPPPTT	EEePPPPTT	EEePPPPTT	EEePPPPTT	---PPPPNN			
**SspH1**	**4NKH_B**	LRR2	LRR3	LRR4	LRR5	LRR6	LRR7			
	**VS sequence**	**TSLPELPEG**	**TSLPSLPQG**	**ASLPTLPPG**	**TSLPEMPPA**	**TSLPALPSG**	**TSLPEMSPG**			
	DSSP-PPII	S---PP-TT	---PPPPTT	S---PPPTT	S--PPPPTT	S--PPPPTT	---PPPPTT			
	PROSS	---PPPPTT	-EEEPPPTT	---PPPPTT	---PPPPTT	---PPPPTT	-EEEPPPTT			
	SENGO	--PPPPp--	--PPPPp--	--pPPPp--	--PPPPp--	--PPPPp--	--pPPPp--			
	XTLSSTR	EEePPPPTT	EEePPPPTT	EEePPPPTT	---PPPPTT	EEePPPPTT	EEePPPPTT			
**SspH1**	**4NKH_C**	LRR2	LRR3	LRR4	LRR5	LRR6	LRR7			
	**VS sequence**	**TSLPELPEG**	**TSLPSLPQG**	**ASLPTLPPG**	**TSLPEMPPA**	**TSLPALPSG**	**TSLPEMSPG**			
	DSSP-PPII	S---PPPTT	---PPPPTT	S---PPPTT	S--PPPPSS	S--PPPPTT	---PPPPTT			
	PROSS	--PPPPPTT	---PPPPTT	---PPPPTT	-EEEPPPTT	---PPPPTT	-EEEPPPTT			
	SENGO	--PPPPp--	--pPPPp--	--pPPPp--	-pPPPPp--	--PPPPp--	--PPPPp--			
	XTLSSTR	--PPPPPTT	EEePPPPTT	EEePPPPTT	EEePPPPNN	EEePPPPNN	EEePPPPTT			
**SspH1**	**4NKH_D**	LRR2	LRR3	LRR4	LRR5	LRR6	LRR7			
	**VS sequence**	**TSLPELPEG**	**TSLPSLPQG**	**ASLPTLPPG**	**TSLPEMPPA**	**TSLPALPSG**	**TSLPEMSPG**			
	DSSP-PPII	SS-PPPPTT	-----PPTT	-S--PPPTT	-S-PPPPTT	-S--PPPTT	---PPPPTT			
	PROSS	---PPPPTT	-EEE-PPTT	---PPPPTT	---PPPPTT	---PPPPTT	---PPPPTT			
	SENGO	-pPPPPp--	--pPPPp--	--PPPPP--	--pPPPp--	--pPPPp--	--pPPPp--			
	XTLSSTR	EEePPPPTT	EEe--PpTT	EEePPPPTT	EEePPPPTT	EEePPPPNN	EEePPpTTT			
**SspH1**	**4NKH_E**	LRR2	LRR3	LRR4	LRR5	LRR6	LRR7			
	**VS sequence**	**TSLPELPEG**	**TSLPSLPQG**	**ASLPTLPPG**	**TSLPEMPPA**	**TSLPALPSG**	**TSLPEMSPG**			
	DSSP-PPII	SS--PPPTT	----PPPTT	S---PPPTT	-S--PPPTT	S--PPPPTT	S--PPPPTT			
	PROSS	-EEEPPPTT	-EEEPPPTT	---P-PPTT	-EEEPPPTT	---PPPPTT	-EEEPPPTT			
	SENGO	-pPPPPp--	-pPPPPp--	--PPPPP--	--PPPPp--	--pPPPp--	--PPPPp--			
	XTLSSTR	EEePPPPTT	EEePPPPTT	EEePPPPTT	EEePPPPTT	EEePPPPTT	EEePPPPTT			
**SspH1**	**4NKH_F**	LRR2	LRR3	LRR4	LRR5	LRR6	LRR7			
	**VS sequence**	**TSLPELPEG**	**TSLPSLPQG**	**ASLPTLPPG**	**TSLPEMPPA**	**TSLPALPSG**	**TSLPEMSPG**			
	DSSP-PPII	S--PPPPTT	---PPPPTT	----PPPTT	S--PPPPTT	S--PPPPTT	S--PPPPTT			
	PROSS	---PPPPTT	---PPPPTT	---PPPPTT	-EEEPPPTT	---PPPPTT	-EEEPPPTT			
	SENGO	--pPPPp--	--PPPPp--	--PPPPp--	--pPPPp--	--PPPPp--	--pPPPp--			

	XTLSSTR	EEePPPPTT	EEePPPPTT	EEePPPPTT	EEePPPPTT	EEePPPPTT	EEePPPPTT				
**SspH1**	**4NKG_C**	LRR2	LRR3	LRR4	LRR5	LRR6	LRR7				
	**VS sequence**	**TSLPELPEG**	**TSLPSLPQG**	**ASLPTLPPG**	**TSLPEMPPA**	**TSLPALPSG**	**TSLPEMSPG**				
	DSSP-PPII	S---PPPTT	-B--PPPTT	SB-PPPPTT	S--PPPPTT	S---PPPTT	S--PP--TT				
	PROSS	-EEEPPPTT	-EEEPPPTT	---PPPPTT	---PPPPTT	-EEEPPPTT	---PP--TT				
	SENGO	--PPPPp--	--PPPPp--	--PPPPp--	--PPPPp--	--PPPPp--	-pPPbBb--				
	XTLSSTR	--PPPPPTT	--PPPPPTT	EEePPPPTT	EEePPPPTT	EEePPPPNN	EEePPpTTT				
**SspH1**	**4NKG_A**	LRR2	LRR3	LRR4	LRR5	LRR6	LRR7				
	**VS sequence**	**TSLPELPEG**	**TSLPSLPQG**	**ASLPTLPPG**	**TSLPEMPPA**	**TSLPALPSG**	**TSLPEMSPG**				
	DSSP-PPII	S--PPPPTT	--PPPPPTT	---PPPPTT	S---PPPTT	SB--PPPTT	SB-----TT				
	PROSS	---PPPPTT	--PPPPPTT	---PPPPTT	----PPPTT	---PPPPTT	-EEEP-PTT				
	SENGO	--PPPPp--	--PPPPp--	--PPPPp--	----PPp--	--PPPPp--	-PPPPp---				
	XTLSSTR	EEePPPPTT	--PPPPPTT	EEePPPPTT	EEePPPPTT	EEePPPPTT	--PPPPPTT				
**IapH3**	**3G06_A**	LRR2	LRR3	LRR4	LRR5	LRR6	LRR7	LRR8	LRR9	LRR10	LRR11
	**VS sequence**	**TSLPALPPE**	**TSLPVLPPG**	**THLPALPSG**	**TSLPVLPPG**	**ASLPALPSE**	**TSLPMLPSG**	**ASLPTLPSE**	**TSLPALPSG**	**TSLPVLPSE**	**TSLPMLPSG**
	DSSP-PPII	S--PPPPTT	S----PPTT	----PPPTT	S--PPPPTT	S--PPPPTT	S--PPPPTT	S---PPPTT	SS-PPPPTT	S---PPPTT	S---PPPTT
	PROSS	---PPPPTT	---P-PPTT	-EEEPPPTT	---PPPPTT	---PPPPTT	---PPPPTT	----PPPTT	---PPPPTT	----PPPTT	----PPPTT
	SENGO	--PPPPp--	--PPPPp--	--PPPPp--	--PPPPp--	--PPPPp--	--PPPPp--	--PPPPp--	-pPPPPp--	--PPPPp--	--PPPPp--
	XTLSSTR	EEePPPPTT	EEePPPPTT	EEePPPPTT	EEePPPPTT	EEePPPPTT	EEePPPPTT	EEePPPPTT	EEePPPPTT	---PPPPTT	EEePPPPTT
**SspH2**	**3CVR_A**	LRR2	LRR3	LRR4	LRR5	LRR6					
	**VS sequence**	**ISLPELPAS**	**STLPELPAS**	**TMLPELPAL**	**TMLPELPTS**	**TFLPELPES**					
	DSSP-PPII	S---PPPTT	S--PPPPTT	S------TT	S--PPPPTT	S---PPPTT					
	PROSS	-EEE-PPTT	-EEE-PPTT	---PP-PTT	---PPPPTT	--PPPPPTT					
	SENGO	--PPPPp--	--PPPPp--	-pPPPPp--	--PPPPp--	--PPPPp--					
	XTLSSTR	EEePPPPTT	EEePPPPTT	EEePPPPTT	---PPPPTT	--PPPPPTT					
**IpaH9.8**	**5B0N_A**	LRR2	LRR3	LRR4	LRR5						
	**VS sequence**	**TNLPELPVT**	**SELPVLPPA**	**ENLPALPDS**	**VSLPSLPQA**						
	DSSP-PPII	S---PPPTT	S---PPPTT	S---PPPTT	----PPPTT						
	PROSS	---PPPPTT	-EEEPPPTT	-EEEPPPTT	----PPPTT						
	SENGO	-pPPPPp--	--PPPPp--	--PPPPp--	----PPp--						
	XTLSSTR	EEePPPPTT	EEePPPPTT	EEePPPPTT	EEePPPPTT						
**IpaH9.8**	**5B0N_B**	LRR2	LRR3	LRR4	LRR5						
	**VS sequence**	**TNLPELPVT**	**SELPVLPPA**	**ENLPALPDS**	**VSLPSLPQA**						
	DSSP-PPII	S---PPPTT	S--PPPPTT	SS--PPPTT	----PPPTT						
	PROSS	---PPPPTT	--PPPPPTT	----P-PTT	----PPPTT						
	SENGO	--PPPPp--	--PPPPp--	--PPPPp--	--PPPPp--						

	XTLSSTR	EEePPPPTT	EEePPPPTT	EEePPPPTT	EEePPPPTT
**IpaH9.8**	**5B0T_A**	LRR2	LRR3	LRR4	LRR5
	**VS sequence**	**TNLPELPVT**	**SELPVLPPA**	**ENLPALPDS**	**VSLPSLPQA**
	DSSP-PPII	S---PPPTT	SS--PPPTT	S---PPPTT	----PPPTT
	PROSS	--PPPPPTT	---PPPPTT	----P-PTT	----PPPTT
	SENGO	-pPPPPp--	-pPPPPp--	-pPPPPp--	-pPPPPp--
	XTLSSTR	EEePPPPTT	EEePPPPTT	EEePPPPTT	EEePPPPTT
**Horse TLR9**	**3WPC_A**	LRR4			
	**VS sequence**	**TTVPALPSS**			
	DSSP-PPII	`SSPPP--TT			
	PROSS	`--PPP-PTT			
	SENGO	`-PPPp----			
	XTLSSTR	`-PPPPPPTT			
	**3WPC_B**	LRR4			
**Horse TLR9**	**VS sequence**	**TTVPALPSS**			
	DSSP-PPII	`SSPPP--TT			
	PROSS	`--PPP-PTT			
	SENGO	`-PPPp----			
	XTLSSTR	`--PPPPPTT			
**Horse TLR9**	**3WPB_A**	LRR4			
	**VS sequence**	**TTVPALPSS**			
	DSSP-PPII	`-SPPP--TT			
	PROSS	`--PPP-PTT			
	SENGO	-pPPpPp--			
	XTLSSTR	`--PPPPPTT			
**Horse TLR9**	**3WPD_A**	LRR4			
	**VS sequence**	**TTVPALPSS**			
	DSSP-PPII	`-SPPP--TT			
	PROSS	`--PPP-PTT			
	SENGO	--PPp----			
	XTLSSTR	`--PPPPPTT			
**Bovine TLR9**	**3WPE_A**	LRR4			
	**VS sequence**	**TTVPALPSS**			
	DSSP-PPII	`-SPPP--TT			
	PROSS	`--PPP-PTT			
	SENGO	-pPPp----			

	XTLSSTR	`-pPPPPPTT
**Mouse TLR9**	**3WPF_A**	LRR4
	**VS sequence**	**TTVPRLPSS**
	DSSP-PPII	`SSPPP--TT
	PROSS	`--PPP-PTT
	SENGO	`-pPbBBb--
	XTLSSTR	`--PPPPPTT
**Mouse TLR9**	**3WPG_A**	LRR4
	**VS sequence**	**TTVPRLPSS**
	DSSP-PPII	`SSPPP--TT
	PROSS	`--PPP-PTT
	SENGO	`-pPbBb---
	XTLSSTR	`--PPPPPNN
**Mouse TLR9**	**3WPI_A**	LRR4
	**VS sequence**	**TTVPRLPSS**
	DSSP-PPII	`SSPPP--TT
	PROSS	`--PPP-PTT
	SENGO	`-pPbBb---
	XTLSSTR	`--PPPPPTT
**Mouse TLR9**	**3WPH_A**	**LRR4**
	**VS sequence**	**TTVPRLPSS**
	DSSP-PPII	SSPPP--TT
	PROSS	--PPP-PTT
	SENGO	-pPPp----
	XTLSSTR	--PPPPPTT
**Fibromodulin**	**5MX0_A**	**LRR1**
	**VS sequence**	**KYLPFVPSR**
	DSSP-PPII	-SPPPPPTT
	PROSS	--PPPPPTT
	SENGO	--PPPPp--
	XTLSSTR	EEePPPPTT
**Fibromodulin**	**5MX0_B**	**LRR1**
	DSSP-PPII	-SPPPPPTT
	PROSS	-EEEPPPTT
	SENGO	--PPPPp--
	XTLSSTR	EEePPPPTT

T-subtype

**SspH1**		LRR1
	**VS sequence**	**TTLPDRLPPH**
**4NKH_A**	DSSP-PPII	----SS--TT
	PROSS	---P---PTT
	SENGO	-pPp-pPp--
	XTLSSTR	EEe-EEeNNN
**4NKH_B**	DSSP-PPII	S---S---TT
	PROSS	---P---PTT
	SENGO	----------
	XTLSSTR	EEe-EEeNNN
**4NKH_C**	DSSP-PPII	S---S---TT
	PROSS	---P---PTT
	SENGO	-pPp-pPp--
	XTLSSTR	EEe---PpNN
**4NKH_D**	DSSP-PPII	S---S---TT
	PROSS	---P--P-TT
	SENGO	-pPp-pPp--
	XTLSSTR	EEe-EEeNNN
**4NKH_E**	DSSP-PPII	S---S---TT
	PROSS	---P-P--TT
	SENGO	----------
	XTLSSTR	EEe-EEeTTT
**4NKH_F**	DSSP-PPII	S---SS--TT
	PROSS	---P---PTT
	SENGO	----------
	XTLSSTR	EEe-EEeNNN
**4NKG_C**	DSSP-PPII	S---S---TT
	PROSS	---P---PTT
	SENGO	-----pPp--
	XTLSSTR	EEe---PpTT
**4NKG_A**	DSSP-PPII	S---SSPPTT
	PROSS	---P--PPTT
	SENGO	-----pPp--
	XTLSSTR	EEe---PpNN
**SspH2**		LRR1
	**VS sequence**	**TTLPDCLPAH**
**3G06_A**	DSSP-PPII	S---S---TT
	PROSS	---P---PTT
	SENGO	-----pPp--
	XTLSSTR	EEe-EEeNNN
**IpaH3**	LRR1	**SSLPDNLPPQ**
**3CVR_A**	DSSP-PPII	S---S---TT
	PROSS	---P---PTT
	SENGO	-----pPp--
	XTLSSTR	EEe---PpNN
**IpaH9.8**		LRR1
	**VS sequence**	**SSLPDNLPAQ**

**5B0N_A**	DSSP-PPII	S---S-PPTT
	PROSS	---P--PPTT
	SENGO	-----pPp--
	XTLSSTR	EEe---PpNN
**5B0N_B**	DSSP-PPII	S---S-PPTT
	PROSS	---P--PPTT
	SENGO	-----pPp--
	XTLSSTR	EEe---PpNN
**5B0T_A**	DSSP-PPII	S---S-PPTT
	PROSS	-EEE--PPTT
	SENGO	-----pPp--
	XTLSSTR	EEe---PpTT
**Horse TLR9**		LRR7
	**VS sequence**	**TTVPRSLPPS**
**3WPC_A**	DSSP-PPII	SSPPSSPPTT
	PROSS	--PPP-PPTT
	SENGO	-pPPp-----
	XTLSSTR	--PPPPPPTT
**3WPC_B**	DSSP-PPII	SSPPSSPPTT
	PROSS	--PPP-PPTT
	SENGO	-pPPp-----
	XTLSSTR	--PPPPPPTT
**3WPB_A**	DSSP-PPII	SS--SSPPTT
	PROSS	--PPP-PPTT
	SENGO	-pPPp-----
	XTLSSTR	EEePPPPPTT
**3WPD_A**	DSSP-PPII	SSPPSSPPTT
	PROSS	--PP--PPTT
	SENGO	-pPPp-----
	XTLSSTR	------PpTT
**Bovine TLR9**		LRR7
	**VS sequence**	**TEVPRRLPPS**
**3WPE_A**	DSSP-PPII	SSPPSSPPTT
	PROSS	--PPP-PPTT
	SENGO	--PPp-----
	XTLSSTR	--PPPPPPTT
**Mouse TLR9**		LRR7
	**VS sequence**	**TKVPRQLPPS**
**3WPF_A**	DSSP-PPII	SSPPSSPPTT
	PROSS	--PPTTPPTT
	SENGO	--PPp-----
	XTLSSTR	--PPp-PpTT
**3WPG_A**	DSSP-PPII	SSPPSSPPTT
	PROSS	--PPTTPPTT
	SENGO	-pPPp-----
	XTLSSTR	--PPp-PpTT
**3WPI_A**	DSSP-PPII	SSPPSSPPTT
	PROSS	--PPTTPPTT
	SENGO	-pPPp-----
	XTLSSTR	--PPp-PpTT

**3WPH_A**	DSSP-PPII	SSPPSSPPTT									
	PROSS	--PPTTPPTT									
	SENGO	-pPPp-----									
	XTLSSTR	--PPp-PpTT									
**SlrP**		LRR1	LRR2	LRR3	LRR4	LRR5	LRR6	LRR7	LRR8	LRR9	LRR10
	**VS sequence**	**TTIPAYIPEQ**	**KSLPENLQGN**	**TSIPATLPDT**	**TELPERLPSA**	**SCLPENLPEE**	**RTLPAHLPSE**	**TALPETLPPG**	**TSLPASLPPE**	**TVLPETLPPT**	**TNLPENLPAA**
**4PUF_A**	DSSP-PPII	S---SB--TT	SSPPS--BS-	----S---TT	----S---TT	----S-PPTT	S-PPS---S-	----S---TT	----S---TT	--PPS-PPTT	----SSPPTT
	PROSS	---P---PTT	-EEEP-P---	-EEE---PTT	--P-TTP-TT	---P--PPTT	--PP-P----	---P----TT	---P---PTT	--PP--PPTT	---PTTPPTT
	SENGO	TSLPELPEG	--PPpbBBb-	-bBb------	--PPp-----	-----pPp--	TSLPELPEG	-EEEPPPTT	0	-pPp------	0
	XTLSSTR	EEe-EEeTTT	--PPPPPEe-	EEe----TTT	EEe---PpTT	EEe---PpTT	--PpEEe---	EEe---PpTT	EEe-EEeTTT	--Pp--PpTT	------PpTT
**4PUF_B**	DSSP-PPII	S----S--SS	-SPPS---S-	--PPS---TT	-S------S-	S---SSPPTT	----S---TT	----S---TT	----S---TT	S-PPS---TT	-SPPSSPPTT
	PROSS	---P--P-TT	--PPP---EE	---P---PTT	P------P--	---P--PPTT	--PP--P-TT	---P-EEETT	---P---PTT	--PP---PTT	--PP--PPTT
	SENGO	---PPPPTT	--PPp---bB	TSLPELPEG	ASLPTLPPG	TSLPEMPPA	-pPp------	TSLPEMPPA	-----pPp--	-pPp-bBb--	-pPp-pPp--
	XTLSSTR	EEe-------	--PPp--EEE	EEe-EEeNNN	EEe-------	EEe---PpTT	EEe---PpTT	----EEeTTT	--Pp--PpTT	--Pp-PPpTT	--Pp--PpTT
**Hman TLR8**		LRR1	LRR4	LRR7							
	**VS sequence**	**QEVPQTVGKY**	**PQIPSGLPES**	**SHVPPKLPSS**							
**3WN4_A**	DSSP-PPII	SSPPS-PPTT	SSPPTTPPTT	SSPPS-PPTT							
	PROSS	--PPTTPPTT	--PPTTPPTT	--PPTTPPTT							
	SENGO	----------	--PPp-----	-pPPp-----							
	XTLSSTR	------PpTT	---Pp-PpTT	EePPPPPPTT							
**3W3J_A**	DSSP-PPII	SSPPTTPPTT	SSPPSSPPTT	SSPPSSPPTT							
	PROSS	--PPTTPPTT	--PPTTPPTT	--PPTTPPTT							
	SENGO	----------	--PPp-----	--PPp-----							
	XTLSSTR	--PpNNPpTT	---Pp-PpTT	---Pp-PpTT							
**3W3J_B**	DSSP-PPII	SSPPS-PPTT	SSPPSSPPTT	SSPPSSPPTT							
	PROSS	---P--PPTT	--PPTTPPTT	--PPTTPPTT							
	SENGO	----------	--PPp-----	--PPp-----							
	XTLSSTR	--Pp---TTT	---Pp-PpTT	---Pp-PpTT							
**3W3N_A**	DSSP-PPII	SSPPTTPPTT	SSPPTTPPTT	SSPPS-PPTT							
	PROSS	---PTTPPTT	--PPTTPPTT	--PPTTPPTT							
	SENGO	----------	-pPPp-----	-pPPp-----							
	XTLSSTR	--PpNNPpTT	--PPp-PpTT	EePPp-PpTT							
**3W3N_B**	DSSP-PPII	SSPPS-PPTT	SSPPTTPPTT	SSPPS-PPTT							
	PROSS	---PTTPPTT	--PPTTPPTT	--PPTTPPTT							
	SENGO	----------	-pPPp-----	-pPPp-----							
	XTLSSTR	--PpNNPpTT	--PPp-PpTT	---Pp-PpTT							
**3W3G_A**	DSSP-PPII	SSPPS-PPTT	SSPPSSPPTT	SPPS-PPTT							
	PROSS	---PTTPPTT	---PTTPPTT	---PTTPPTT							
	SENGO	----------	--PPp-----	-pPPp-----							
	XTLSSTR	--PpNNPpTT	--PPp-PpTT	EePPPPPPTT							
**3W3G_B**	DSSP-PPII	SSPPS-S-TT	SSPP--PPTT	SSPPSSPPTT							
	PROSS	--PPTTP---	--P-P-PPTT	--PPTTPPTT							
	SENGO	-pPp------	--PPp-----	-pPPp-----							
	XTLSSTR	--PpNN-TTT	-------TTT	EePPPPPPTT							
**3W3K_A**	DSSP-PPII	SSPPS-PPTT	SSPPSSPPTT	SSPPSSPPTT							
	PROSS	--PPTTPPTT	---PTTPPTT	--PPTTPPTT							
	SENGO	----------	--PPp-----	--PPp-----							
	XTLSSTR	--PpNNPpTT	---Pp-PpTT	--PPp-PpTT							
**3W3K_B**	DSSP-PPII	SSPPS---SS	SSPPSSPPTT	SSPPSSPPTT							
	PROSS	--PPTTP---	--PPP-PPTT	--PPTTPPTT							

	SENGO	----------	--PPp-----	--PPp-----
	XTLSSTR	--PpNNPpNN	---PPPPPNN	---Pp--TTT
**3W3L_A**	DSSP-PPII	SSPPTTS-TT	SSPPSSPPTT	SSPPSSPPTT
	PROSS	--PPTTPPTT	--PPTTPPTT	--PPTTPPTT
	SENGO	----------	-pPPp-----	--PPp-----
	XTLSSTR	--PpNNPpTT	EEePp-PpNN	--PPPPPPTT
**3W3L_B**	DSSP-PPII	SSPPSSPPTT	SSPPSSPPTT	SSPPSSPPTT
	PROSS	--PPTTPPTT	--PPTTPPTT	--PPTTPPTT
	SENGO	----------	-pPPp-----	--PPp-----
	XTLSSTR	--PpNNPpTT	---Pp-PpTT	--PPp-PpTT
**3W3L_C**	DSSP-PPII	SSPPS-PPTT	SSPPSSPPTT	SSPPSSPPTT
	PROSS	--PPTTPPTT	--PPTTPPTT	--PPTTPPTT
	SENGO	----------	--PPp-----	--PPp-----
	XTLSSTR	--PpNNPpNN	---PPPPPNN	--PPPPPPTT
**3W3L_D**	DSSP-PPII	SSPPS-PPTT	SSPPSSPPTT	SSPPSSPPTT
	PROSS	--PPTTPPTT	--PPTTPPTT	--PPTTPPTT
	SENGO	----------	-pPPp-----	--PPp-----
	XTLSSTR	--PpNNPpTT	---Pp-PpNN	--PPPPPPTT
**3W3M_A**	DSSP-PPII	SS--S-PPTT	SSPPSSPPTT	SSPPSSPPTT
	PROSS	---PTTPPTT	--PPTTPPTT	--PPTTPPTT
	SENGO	----------	-pPPp-----	--PPp-----
	XTLSSTR	--PpNNPpTT	--PPPPPPNN	---PPPPPTT
**Bovine decorin**		LRR1	LRR4	LRR7
	**VS sequence**	**EKVPKDLPPD**	**KELPEKMPKT**	**TTIPQGLPPS**
**1XEC_A**	DSSP-PPII	SSPP-SPPTT	SBPPSS--TT	-SPPSSPPTT
	PROSS	--PP--PPTT	--PPP-P-TT	--PPTTPPTT
	SENGO	----------	--PPp-----	-pPPp-----
	XTLSSTR	--PPp-PpTT	--PPPPPPTT	--PPp-PpTT
**1XEC_B**	DSSP-PPII	SSPP-SPPTT	SBPPSS--TT	-SPPSSPPTT
	PROSS	--PP--PPTT	-EEETTP-TT	--PPTTPPTT
	SENGO	----------	--PPp-----	-pPPp-----
	XTLSSTR	--Pp--PpTT	--PPp-PpTT	--PPp-PpTT
**1XCD_A**	DSSP-PPII	SSPP-SPPTT	SBPPSSPPTT	-SPPSSPPTT
	PROSS	--PP--PPTT	--PPP-PPTT	--PPTTPPTT
	SENGO	----------	--PPp-----	-pPPp-----
	XTLSSTR	--PPp-PpTT	--PPPPPPTT	--PPPPPPTT
**1XKU_A**	DSSP-PPII	-SPPPSPPTT-	SBPPSSPPTT	-SPPSSPPTT
	PROSS	--PPTTPPTT	--PPTTPPTT	--PPTTPPTT
	SENGO	TTVPRLPSS	--PPp-----	-pPPp-----
	XTLSSTR	--PPp-PpTT	--PPp-PpTT	--PPp-PpTT
**Bovine biglycan**		LRR1	LRR4	LRR7
	**VS sequence**	**KAVPKEISPD**	**VEIPPNLPSS**	**TGIPKDLPET**
**2FT3_A**	DSSP-PPII	SSPPS---TT	-SPPSS--TT	SSPPSSS-SS
	PROSS	--PPP----T	P-PPTTP---	--PP---PTT
	SENGO	TTVPRLPSS	--PPp-----	TTVPRLPSS
	XTLSSTR	--Pp---TTT	--Pp--PpTT	--Pp--PpNN
**2FT3_B**	DSSP-PPII	SSS-S---TT	-SPPSSPPTT	SSPPSSS-TT
	PROSS	--PP---PTT	--PP--PPTT	--PP--PPTT
	SENGO	TTVPRLPSS	--PPp-----	TTVPRLPSS
	XTLSSTR	-------TTT	--Pp--PpTT	--Pp--PpTT

**2FT3_C**	DSSP-PPII	SSPPS---TT	-SPPSSPPTT	SSPPSSS-TT
	PROSS	--PP---PTT	--PPTTPPTT	--PP---PTT
	SENGO	--PPp-----	--PPp-----	TTVPRLPSS
	XTLSSTR	-------TTT	---Pp-PpTT	--Pp--PpTT
**2FT3_D**	DSSP-PPII	SSS-S---TT	-SPPSSPPTT	SSPPSSS-TT
	PROSS	--PP---PTT	--PPTTPPTT	--PP---PTT
	SENGO	TTVPRLPSS	--PPp-----	TTVPRLPSS
	XTLSSTR	-------TTT	--PPp-PpTT	--Pp--PpTT
**2FT3_E**	DSSP-PPII	SSPPS---TT	-SPPSSPPTT	SSPPSSS-TT
	PROSS	--PP---PTT	--PPTTPPTT	--PP---PTT
	SENGO	TTVPRLPSS	--PPp-----	TTVPRLPSS
	XTLSSTR	-------TTT	---Pp-PpTT	---Pp-PpTT
**2FT3_F**	DSSP-PPII	SSPPS---TT	-SPPSSPPTT	SSPPSSS-TT
	PROSS	--PP----TT	--PPTTPPTT	--PP---PTT
	SENGO	TTVPRLPSS	--PPp-----	TTVPRLPSS
	XTLSSTR	-------TTT	---Pp-PpTT	--Pp--PpTT
**Fibromodulin**		LRR4	LRR7	
	**VS sequence**	**TRMPGPLPRS**	**RKVPDGLPSA**	
**5MX0_A**	DSSP-PPII	-SPP----TT	SSPPS---TT	
	PROSS	--PP---PTT	--PP---PTT	
	SENGO	-bBBbpPp--	-pPp------	
	XTLSSTR	--Pp--PpTT	--Pp---NNN	
**5MX0_B**	DSSP-PPII	-SPP----TT	SSPPSS--TT	
	PROSS	--PP---PTT	--PP---PTT	
	SENGO	-bBBbpPp--	-pPp------	
	XTLSSTR	--Pp---TTT	--Pp---NNN	
**Human FLRT2**		LRR1	LRR3	LRR6
	**VS sequence**	**TSVPLGIPEG**	**DEFPMNLPKN**	**SSVPVGLPVD**
**4V2D_A**	DSSP-PPII	SSPPS---SS	-BPPSSPPTT	SBPPS---TT
	PROSS	--PP---PTT	--PPTTPP--	--PP---PTT
	SENGO	-pPp------	-pPPp-----	----------
	XTLSSTR	--PpEEe---	--PPp-PpTT	--PpEEeNNN
**Mouse FLRT3**		LRR1	LRR3	LRR6
	**VS sequence**	**TSIPVGIPED**	**DEFPTNLPKY**	**STIPGGLPRT**
**4V2E_A**	DSSP-PPII	SSPPS---SS	-BS-SSPPSE	SSPPS---TT
	PROSS	--PP---P--	--PP--PP--	--PP-EEETT
	SENGO	-pPp------	--PPp-----	-----bBb--
	XTLSSTR	--PpEEe-EE	--PPp-Pp--	--PpEEeNNN
**4V2E_B**	DSSP-PPII	SSPPS---S-	-SS-SSPPSE	SSPPS---TT
	PROSS	--PP---P-P	--PPTTPP--	--PP---PTT
	SENGO	-pPp------	--PPp-----	0
	XTLSSTR	--PpEEe---	--PPp-Pp--	--PpEEeTTT
**Human FLRT3**		LRR1	LRR3	LRR6
	**VS sequence**	**TSIPTGIPED**	**DEFPTNLPKY**	**STIPWGLPRT**
**5CMP_A**	DSSP-PPII	SSPPS-PPTT	-BPP-SPPTT	SBPPS---TT
	PROSS	--PP--PPTT	--PP--PPTT	--PP---PTT
	SENGO	0	0	0
	XTLSSTR	EEe---PpTT	--PPp-PpTT	EEe-EEeTTT
**5CMP_B**	DSSP-PPII	SSPPS-PPTT	-BPP-S--TT	SBPPS---TT
	PROSS	--PP--PPTT	--PPTTPPTT	--PP---PTT

	SENGO	0	0	-----bBb--
	XTLSSTR	EEe---PpTT	--PPp-PpTT	EEe-EEeTTT
**5CMP_C**	DSSP-PPII	SSPPS-PPTT	EEe---PpTT	SSPPS---TT
	PROSS	--PP--PPTT	--PPTTPPTT	--PP---PTT
	SENGO	S---S---TT	LRR1	0
	XTLSSTR	EEe---PpTT	--PPp-PpTT	EEe-EEeTTT
**5CMP_D**	DSSP-PPII	SSPPS-PPTT	-BPP-SPPTT	SSPPS---TT
	PROSS	--PP--PPTT	--PP--PPTT	--PP---PTT
	SENGO	S---SSPPTT	S---SSPPTT	-----bBb--
	XTLSSTR	EEe---PpTT	--PPp-PpTT	EEe-EEeTTT
**Mouse FLRT3**		LRR1	LRR3	LRR6
	**VS sequence**	**TSIPVGIPED**	**DEFPTNLPKY**	**STIPGGLPRT**
**4YEB_A**	DSSP-PPII	SSPPS---TT	BSS--S--TT	SB--S-PPTT
	PROSS	-PPP---PTT	--PP---PTT	--P---PPTT
	SENGO	-PPp------	0	------PPp-
	XTLSSTR	-PPpEEeNNN	p-Pp---TTT	------PPp-
**Mouse FLRT2**		LRR1	LRR3	LRR6
	**VS sequence**	**TSVPLGIPEG**	**DEFPMNLPKN**	**SSVPVGLPVD**
**5FTT_F**	DSSP-PPII	SSPPS---SS	SBPPSSPPTT	SSPPSSPPTT
	PROSS	--PP---PTT	--PPTTP---	--PP--PPTT
	SENGO	0	-PPPp-----	0
	XTLSSTR	--PpEEeNNN	PPPPP-PpTT	--Pp--PpNN
**5FTT_B**	DSSP-PPII	SSPPS---TT	SBPPSSPPTT	SSPPSSPPTT
	PROSS	--PP---PTT	--PP--P---	--PP--PPTT
	SENGO	----------	-pPPp-----	0
	XTLSSTR	--PpEEeNNN	PPPPP-PpTT	--Pp--PpNN

A one letter code is used to represent a specific conformation; P and p, PPII; E and e, β-strand; T, β-turn; N, non-hydrogen-bonded β-turn; H, α-helix; and S, bend.
